# RNA sequencing of identical twins discordant for autism reveals blood-based signatures implicating immune and transcriptional dysregulation

**DOI:** 10.1186/s13229-019-0285-1

**Published:** 2019-11-07

**Authors:** Ayden Saffari, Matt Arno, Eric Nasser, Angelica Ronald, Chloe C. Y. Wong, Leonard C. Schalkwyk, Jonathan Mill, Frank Dudbridge, Emma L. Meaburn

**Affiliations:** 1 Department of Non-communicable Disease Epidemiology, London School of Hygiene and Tropical Medicine, London, UK; 2 Centre for Brain and Cognitive Development, Department of Psychological Sciences, Birkbeck, University of London, London, UK; 3 Edinburgh Genomics, University of Edinburgh, Edinburgh, Scotland UK; 4 King’s Genomics Centre, King’s College London, London, UK; 5 Social Genetic and Developmental Psychology, Institute of Psychiatry, Psychology and Neuroscience, King’s College London, London, UK; 6 School of Life Sciences, University of Essex, Colchester, UK; 7 University of Exeter Medical School, University of Exeter, Exeter, UK; 8 Department of Health Sciences, University of Leicester, Leicester, UK

**Keywords:** Autism spectrum disorder, Immune, MZ twins, RNA-seq, Gene expression, DNA methylation, Transcriptomics, Epigenomics, Discordance

## Abstract

**Background:**

A gap exists in our mechanistic understanding of how genetic and environmental risk factors converge at the molecular level to result in the emergence of autism symptoms. We compared blood-based gene expression signatures in identical twins concordant and discordant for autism spectrum condition (ASC) to differentiate genetic and environmentally driven transcription differences, and establish convergent evidence for biological mechanisms involved in ASC.

**Methods:**

Genome-wide gene expression data were generated using RNA-seq on whole blood samples taken from 16 pairs of monozygotic (MZ) twins and seven twin pair members (39 individuals in total), who had been assessed for ASC and autism traits at age 12. Differential expression (DE) analyses were performed between (a) affected and unaffected subjects (*N* = 36) and (b) within discordant ASC MZ twin pairs (total *N* = 11) to identify environmental-driven DE. Gene set enrichment and pathway testing was performed on DE gene lists. Finally, an integrative analysis using DNA methylation data aimed to identify genes with consistent evidence for altered regulation in *cis*.

**Results:**

In the discordant twin analysis, three genes showed evidence for DE at FDR < 10%: *IGHG4*, *EVI2A* and *SNORD15B*. In the case-control analysis, four DE genes were identified at FDR < 10% including *IGHG4*, *PRR13P5*, *DEPDC1B*, and *ZNF501*. We find enrichment for DE of genes curated in the SFARI human gene database. Pathways showing evidence of enrichment included those related to immune cell signalling and immune response, transcriptional control and cell cycle/proliferation. Integrative methylomic and transcriptomic analysis identified a number of genes showing suggestive evidence for *cis* dysregulation.

**Limitations:**

Identical twins stably discordant for ASC are rare, and as such the sample size was limited and constrained to the use of peripheral blood tissue for transcriptomic and methylomic profiling. Given these primary limitations, we focused on transcript-level analysis.

**Conclusions:**

Using a cohort of ASC discordant and concordant MZ twins, we add to the growing body of transcriptomic-based evidence for an immune-based component in the molecular aetiology of ASC. Whilst the sample size was limited, the study demonstrates the utility of the discordant MZ twin design combined with multi-omics integration for maximising the potential to identify disease-associated molecular signals.

**Electronic supplementary material:**

The online version of this article (10.1186/s13229-019-0285-1) contains supplementary material, which is available to authorized users.

## Background

Autism spectrum condition (ASC) is a neurodevelopmental disorder typified by deficits in social communication and by stereotyped behaviours. Twin, family and DNA-based studies consistently indicate a strong genetic basis for the disorder, with both common and rare variants contributing to overall risk [1–6]. While a significant proportion of genetic liability for ASC can now be captured by polygenic profile scores derived from large-scale genome-wide association studies [1, 7], there exists a gap in our ability to build mechanistic models of how genetic and non-genetic risk factors influence early brain development and result in autism symptoms. Complementary functional genomic approaches that profile gene expression and epigenetic regulation could prove valuable in this regard. Because these intermediate molecular traits lie between genotype and phenotype, they can be informative about underlying mechanisms even when the trait is multifactorial and highly genetically heterogeneous, by providing evidence of convergence of risk on common downstream pathways [8]. In addition, gene regulatory and expression signatures may also have utility as biomarkers [9] as they can display individual level variability that is not completely heritable [10–12], may persist long after the initial exposure [13] and may be detectable in tissues other than those primarily affected [14, 15].

A number of studies have characterised global gene expression in ASC, typically using microarrays and whole blood or blood-derived cells. A recent mega-analysis of seven primary blood-based microarray datasets extended previous array-based meta-analyses [16, 17] and found support for reduced-expression of transcripts relating to innate and adaptive immunity [18]. Brain-based transcriptomic investigations of ASC using arrays are scarcer and generally limited to examination of post-mortem brain regions across a wide age range [19, 20], some of which have recently been meta-analysed to populate a searchable differential gene expression database for ASC [21].

More recently, gene expression studies have utilised RNA-seq as it can interrogate gene expression in much greater resolution than expression arrays, including low abundance transcripts as well as alternatively spliced, and non-coding regulatory transcripts [22]. RNA-seq has been used to characterise genome-wide patterns of expression for a range of neurodevelopmental and psychiatric conditions including major depressive disorder (MDD) [23], schizophrenia [24], bipolar disorder [25] and Alzheimer’s disease [26]. RNA-seq has also been used to investigate gene expression in a developmental context in healthy controls [27], and also in ASC-affected individuals [19, 28]. The largest published autism RNA-seq study of post-mortem brain tissue found evidence for cortex-specific differential gene expression and alternative splicing events, with enrichment for genes expressed in microglia and astrocytes [29]. A second (smaller) study identified an ASC-associated gene co-expression module that was enriched for microglia cell states and type 1 interferon pathway, and the authors hypothesised a putative link between neuronal activity-dependent gene regulation mediated by innate immune response [28].

To date, the majority of gene expression studies of ASC have utilised case-control designs that are limited in their capacity to investigate the influence of environmental factors, due to the potential for genetic and other sources of confounding. Intermediate molecular traits such as DNA methylation and transcription can be influenced by genetic variation (i.e. mQTLs and eQTLs), in addition to displaying environmentally linked and stochastic variability, complicating attempts to separate genetically driven from environmentally driven effects (and their interactions) [10]. To overcome some of these challenges, a number of authors have recommended a discordant identical twin-based design [30, 31]. Because identical (monozygotic; MZ) twins are matched for age, sex, genetic background, as well as family-wide factors [32], divergence in gene expression patterns or epigenetic marks can indicate causes or consequences of disease that are independent of genetic effects [33]. In ASC, despite the high heritability, MZ concordance rates do not rise above 90% [34], suggesting a role for non-shared effects such as epigenetic, gene expression, other environmental and/or stochastic factors [35, 36]. Epidemiological investigation of what these specific non-shared environmental factors might be is difficult, especially given that the relevant exposures are likely to occur early in development. Investigation of the intermediate molecular phenotype of gene expression might therefore act as a useful proxy for relevant non-shared environmental effects as it is tangibly linked to underlying cellular processes and can reflect the aggregate effects of environmental influences that could persist long after the initial exposure. Identification of environmentally attributable differential gene expression would provide critical insights into the mechanistic developmental origins of ASD, as well as provide a potential biomarker for diagnostics, treatment assessment or intervention studies.

Genome-wide gene expression profiling of discordant MZ twins has previously been performed for a range of cognitive, physical, psychiatric and neurological traits including BMI [37, 38], intelligence [39], bipolar disorder [40] and Alzheimer’s disease [41]. There has however been limited application of this methodology to ASC. A study by Hu et al. examined gene expression in three pairs of MZ ASC discordant and two pairs of MZ ASC concordant twins and identified expression differences in gene networks involved in neurological function, nervous system development and inflammation [42]. Several studies have successfully utilised MZ twins to investigate altered DNA methylation in psychiatric traits [43–46]. We previously performed genome-wide DNA methylation profiling in the cohort used in the current study that included ASC discordant, ASC concordant, as well as unaffected concordant MZ twin pairs, which investigated differences within discordant pairs and between groups (cases and controls) [47]. While ASC was not associated with global changes in DNA methylation within-discordant pairs, a number of CpG sites were found to be differentially methylated between co-twins, with the top association a CpG site located in the promoter of the *NFYC* gene.

As far as we are aware, there are no previous studies that have utilised a MZ differences twin design to characterise genome-wide gene expression and DNA methylation differences in the same individuals. Here, we conduct a genome-wide gene expression study using RNA-seq to profile global patterns of gene expression in subjects from a cohort of ASC discordant, ASC concordant and unaffected concordant control MZ twin pairs for whom we have previously characterised DNA methylation [47]. Genome-wide gene expression profiles were generated and two main analyses carried out. Firstly, we performed within-group analysis of the discordant ASC twins with the aim to identify genes and pathways showing altered expression in common in ASC compared to non-ASC co-twins, attributable to non-shared environmental factors. Secondly, we performed a case-control analysis by comparing ASC affected from both discordant and concordant groups with unaffected, age-matched controls to identify genes and pathways commonly disrupted in ASC, which could be attributable to both genetic and/or environmental factors. Finally, we performed data integration with previously published methylation array data to determine if identified gene expression signatures showed any relation to previously identified DNA methylation signatures, and whether such an integrative analysis could lend further support to identified pathways and mechanisms.

## Methods

### Subjects

Subjects were originally recruited as part of the Twins Early Development Study (TEDS; https://www.teds.ac.uk/), a longitudinal study investigating the cognitive and behavioural development of twins born in England and Wales between January 1994 and December 1996 [48–50]. TEDS participants completed various web and telephone-based tests and questionnaires at regular intervals over childhood and adolescence designed to assess various aspects of cognition, language and behaviour (see [50] for further details). Twins were assessed for ASC-related traits and behaviours at ages 8 and 12 using the Childhood Autism Spectrum Test (CAST). The CAST is a 31-item questionnaire that assesses ASC traits [51, 52]. The 31 items are combined additively to give a total CAST score out of 31, with those scoring ≥ 15 categorised as “at risk” of having an ASC. Individuals identified as at risk were also formally assessed at home using the Autism Diagnostic Interview-Revised (ADIR) [53] and the Autism Diagnostic Observation Schedule (ADOS) [54] both considered gold standard diagnostic tools. Twins were selected from the TEDS sample of ~ 10,000 twin pairs based on scores on the CAST total score or whether a clinical diagnosis of ASC had been made, and assigned to three study groups: concordant ASC (MZ pairs where both members of the twin pair had a formal ASC diagnosis; 28 pairs identified, of which 25 male), discordant ASC (MZ pairs where one member of the twin pair had formal ASC diagnosis; 14 pairs identified, 11 male) and low total CAST controls (unaffected MZ pairs where both twins scored less than or equal to the sample mean in total CAST score (≤ 6); 120 pairs identified, of which 45 were male). We additionally required that the concordant MZ twin pairs with low ASC symptoms had birth weights within 1 SD of each other, had no reported medical conditions and were not perinatal outliers. All samples were of white ethic origin. Identified families were contacted and invited to participate in the study, which resulted in a total of 23 MZ twin pairs visiting the SGDP lab to have whole blood samples collected by a trained phlebotomist: six concordant ASC, six discordant ASC and 11 control pairs (total *N* = 46). Blood samples were successfully obtained for 40 individuals: ten concordant ASC (four pairs, two individual members of a twin pair), 11 discordant ASC (five pairs, one individual), 19 concordant low total CAST (unaffected controls) (eight pairs, three individuals).

### RNA isolation, library preparation and sequencing

**Fig. 1:**
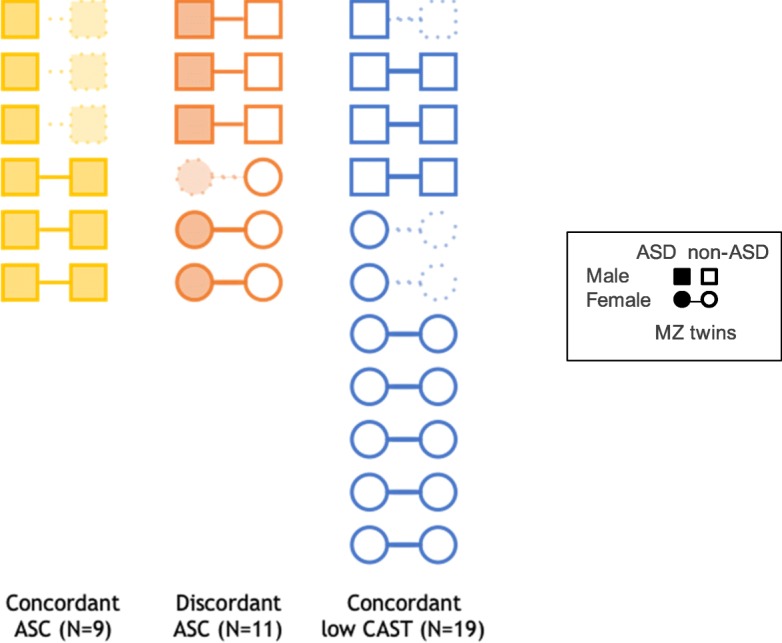
Study group sample characteristics (*N* = 39)

Whole blood samples were collected and stored using the PAXgene system (PreAnalytiX QIAGEN, Germany). Total RNA was extracted (5 μg for each sample) and the Ribo-Zero Globin kit (Ambion, USA) was used to deplete ribosomal and globin RNAs. Sample concentration and quality was assessed using the RNA 6000 Nano LabChip and Agilent 2100 BioAnalyzer systems (Agilent Technologies, USA). The majority of samples (36/40) had RNA integrity (RIN) values between 7 and 9 (mean = 8.2). The four remaining samples had RIN values < 7 (mean = 4.8), with one sample from the concordant ASC group found to be severely degraded (RIN = 2.6) which was subsequently excluded from further analysis (see Fig. 1). Stranded total RNA libraries were prepared for the remaining 39 samples using the Illumina TruSeq Stranded Total RNA Library Prep Kit v3 (Illumina, USA) following the manufacturer’s protocol for a low-throughput experiment. The final libraries consisted of indexed fragments with median length 326 bp (160 bp inserts). The Agilent BioAnalser system was used to assess the fragment length distribution. The libraries were then pooled and randomised across the lanes of a single flow cell of the Illumina HiSeq 2000 (Illumina, USA) sequencing in 100-bp paired end mode. Sample libraries were randomised across lanes in an approximation of a balanced design (to the extent that was possible with the starting sample characteristics), with twin pairs split across the lanes and each lane containing subjects from each of the three study groups (discordant ASC, concordant ASC and concordant controls). To ensure each lane had the same number of samples and read density, one sample from the concordant ASC group was run in duplicate, so that in total RNA libraries for 40 samples were sequenced (for 39 individuals). The resulting reads were demultiplexed by filtering according to the 6 bp index sequences contained within the adaptors and unique to each sample.

### Data preprocessing, mapping and quantification

The fastq files containing the sequenced reads for each sample were downloaded and initial data preprocessing and quality control were carried out. The FastQC program (v0.11.2) [55] was used to produce general sequencing metrics. The number of reads per library ranged between 38 and 78 M, with a mean of approximately 54 M reads per sample. Plots for per-base sequence quality, per-sequence quality, sequence length distribution, GC content were inspected, and found to be as expected for all samples. The average sequence duplication rate for each library was around 0.21, also in line with expectations [56]. Next, to improve mapping efficiency, the cutadapt tool (v1.4.1, [57]) was used to trim lower quality base pair calls from the ends of sequences, and to remove any reads mapping to Illumina adaptor sequences.

Reads were mapped to the Ensembl hg19 reference genome using Tophat2 (v2.0.12) [58]. To assess the effectiveness of read mapping and the quality of the alignments, RNA-SeQC (v1.1.8) [59] was used to produce summary statistics including total proportion of mapped reads, proportion of reads mapping in pairs, the effectiveness of the stranded protocol, fraction of reads mapping to exonic or intronic regions and coverage along the length of the transcripts. These metrics were compared to guidelines from GEUVADIS [56]. The overall indications were that the sequencing data generated was of a high quality, with on average 78% of total reads mapping in their forward and reverse read pairs, 47% of these reads originating from exonic regions, a mean coverage of 6× for less abundant transcripts (bottom 1000) and 732× for transcripts present at higher levels (top 1000).

The featureCounts program [60] from the subread package (v1.4.6) [61] was next used to summarise and quantify the mapped reads, by counting the number of reads overlapping Ensembl gene annotations. Finally, filtering was performed to remove genes with low counts with required counts of ≥ 1 CPM (counts per million) in at least three samples leaving count data for 17,765 genes.

### Post quantification QC

Following quantification, a number of QC steps were performed to assess the quality of the final gene expression profiles and identify any outliers. Firstly, concordance between expression profiles within the twin pairs was examined. As gene expression is at least partially heritable (mean h^2^ ~ 0.14 to 0.3) [11, 62], twins would be expected to show higher correlation in gene expression profiles than in comparison to unrelated subjects. To confirm this, counts were normalised by total library size and transformed into logCPM (log counts per million), and Pearson’s correlation was calculated. A heatmap was produced to visualise the results, and mean correlation values within pairs and between unrelated subjects were recorded. Next, clustering and data reduction techniques were used to produce visualisations for examining the similarity of the expression profiles and determine if any clustering by technical or biological factors was observed, specifically by case status, study group, sex or flow cell lane. Firstly, hierarchical, unsupervised clustering was performed with the hclust function in R using Euclidean distance between samples based on gene expression profiles. These were plotted as dendrograms (Additional file 1: Figure S1). Multi-dimensional scaling (MDS) plots were also generated for further visualisation of sample similarity based on leading log fold changes between pairs of samples (Additional file 1: Figure S2).

### Differential gene expression

The edgeR package [63] was used to perform differential expression (DE) analyses. This uses a generalised linear modelling (GLM) strategy to model systematic and biological sources of variation, and an empirical Bayes method to improve estimates by shrinking gene-wise dispersions towards the common trend. Following the standard edgeR workflow, count data was normalised for total library size, using trimmed mean of *M* values (TMM), which minimises log FCs between samples for the majority of genes. To test for differential expression attributable to ASC case status, for each gene a negative binomial GLM model was used and a likelihood ratio test (LRT) performed. Multiple testing was controlled at FDR 10%, chosen due to the discovery nature of the study.

Two primary DE analyses were performed. Firstly, a within group analysis of the ASC discordant twins was performed to identify differences in gene expression associated with ASC which could be attributable to non-shared environment. This analysis will be referred to as “discordant group” from this point. The model used specified gene count as the outcome which was regressed on pair—a categorical variable representing twin-pair membership, and case—a binary variable denoting case status. The results were further annotated to indicate whether the direction of effect (log FC) was consistent across pairs. For the second analysis, cases and controls from across the complete sample were compared to identify gene expression differences associated with ASC that could be genetic and/or environmental in origin. We refer to this analysis and the results as “case-control” from here on. The model used was the same as used in the discordant twin analysis above, with gene count the outcome and pair and case status the explanatory variables.

Finally, within-pair gene expression differences were assessed to determine whether these showed consistent differences in magnitude or overall distribution between the three study groups. For each pair separate, edgeR models were fit (comparing one co-twin to the other). Then for each of the study groups, absolute logFCs across all genes and pairs were recorded, and scatter plots produced. Both *t* tests and Kolmogorov–Smirnov (KS) tests were performed comparing the means and overall distributions for each group (Additional file 1: Figure S5).

### Gene set enrichment and pathways

Gene set enrichment testing was performed to investigate whether identified differential expression signals involved known ASC risk genes. The geneSetTest method from limma [64] was used with ranks only, comparing the mean rank of the set of interest with permuted sets. Three different ASC genesets were tested for enrichment. The first of these was from the Simons Foundation Autism Research Initiative (SFARI) gene resource [65, 66], a manually curated reference database of ASC genes from published molecular studies including candidate genes, known common and rare variants and copy number variants linked to ASC. These are divided into different categories based on the strength of evidence. We combined these into two gene sets: category 1 and 2 (high confidence and strong candidate) (*n* = 91), and categories 3, 4 and 5 (suggestive evidence, minimal evidence and hypothesised) (*n* = 842). The second gene set was derived from the association results of the iPSYCH-PGC ASD GWAS meta-analysis of over 18,381 individuals with ASD [67], setting a *p* value threshold of 1 × 10^−6^ and taking the set of genes that these mapped to (according to Ensembl hg19 annotation) (*n* = 28). Finally, we used the TADA set of genes from an exome sequencing study of 2270 trios, which identified genes showing evidence for increased burden of de-novo loss of function in autistic subjects (*n* = 107) [2]. All sets were accessed and downloaded in July 2019.

Pathway enrichment analyses were carried out to identify biological pathways and cellular processes over-represented within the differentially expressed genes. The ROMER method from limma was used. Pathway datasets were from the Molecular Signatures Database (MSigDB) [68], comprising biochemical pathways (from Kyoto Encyclopedia of Genes and Genomes—KEGG [69] and REACTOME [70]), gene families (from Gene Ontology—GO [71]) and various other sources of annotation. Two sets were used, the C2 curated set—containing 4726 canonical metabolic and signalling pathways, and disease gene expression signatures, and the H1 hallmark set—50 gene sets representing well-defined biological states or processes where genes show coordinated expression.

### Integration with DNA methylation data

Using an existing Illumina 27 K DNA methylation dataset generated on the same sample [47], an integrative analysis was performed to derive further insights into the functional relevance of DE genes and identify genes with consistent evidence for altered regulation in ASC, representing putative disorder associated *cis* expression quantitative trait methylation loci (eQTMs). The stored Beta values were first converted to *M* values as these have distributional properties more suitable for use in standard linear regression [72], and differential methylation analysis performed. The ComBat function from the SVA package [73] was used to identify batch and other unmeasured sources of variation—in this case, none were identified. Next, edgeR [74] was used to fit a linear model for discordant group and case-control comparisons, using the same models described above for the RNA-seq data. Given the limited of coverage of the array, a simple gene-level integration strategy was followed looking for evidence of *cis* regulatory signals across the datasets. Firstly, to assess the level of concordance, Spearman’s correlation coefficient was calculated between gene-level expression (as normalised counts per million) and gene-level DNA methylation (aggregating CpG probes and taking the median Beta value as representative). Secondly, evidence combination was performed. The SLK method from comb-p [75] was used to obtain spatial correlation adjusted *p* values for each CpG site, setting the distance parameter to 2000 bp and region filter to 0.05. These were summarised at the gene level by taking the minimum *p* value. This was then combined with the gene level statistics from the RNA-seq results using empirical Brown’s method [76], which takes into account correlation between measures (using the assay data), to derive a combined *p* value for genes measured in both assays. The final list of genes was then sorted by combined *p* value and the top 20 retained.

### Male subgroup analysis

Previous studies have identified sex-specific patterns of gene expression associated with ASC [77, 78], with the latter also demonstrating that separate analyses of males and females improved the ability to detect ASC-related differences. Here, because the sample contained only two affected females (both discordant group), we were not adequately powered to perform a sex-stratified analysis. Instead, the main analyses were rerun using male subjects as an explicit control for sex, which in the main analysis was presumed to have been captured by pair status (as a nested effect—since MZ twins are the same sex).

QQ plots were generated and inspected (data not shown) to compare the models in terms of overall distribution of *p* values. The lists of top differentially expressed genes were also compared to evaluate the impact of excluding female samples on the ranks of FDR significant DE genes and whether this improved our ability to detect ASC-related differences. See Additional file 1: Table S13 and S14.

### Blood cell composition and surrogate variable analysis

Issues related to cellular heterogeneity have received much attention in the transcriptomics [79–83] and epigenomics literature [84–88]. Cell composition can act as a confounder, or alternatively represent a variable of interest if disease-associated changes in cell populations or cell subtype-specific expression patterns are disease relevant. In the context of this study, it was not clear how cellular heterogeneity should be dealt with since changes in cell composition could well be ASC relevant, with previous studies finding evidence for increased numbers of activated B and NK cells in ASC affected individuals [89, 90]. As full blood cell counts for our samples were not available, we performed cell subtype estimation as a post hoc analysis, to determine whether the main findings were likely being driven by differences in blood cell composition. The XCell software was used to infer cell counts, which compares the experimental RNA-seq data to expression signatures spanning 64 immune and stromal cell types and calculates an enrichment score [83]. Blood cell adjustment was performed as part of surrogate variable analysis.

Surrogate variable analysis (SVA) [73] was performed firstly to identify any unmodelled technical variation and secondly to reduce the number of parameters required to model inferred blood cell counts—since these could be much greater than the sample size. The two main analyses and the male-only versions were re-run using models including surrogate variable adjustment. Correlation between identified SVs and cell counts were calculated to determine if these were likely to be accounting for these variables. As before, QQ plots (not shown) and relevant summary statistics were generated and inspected to compare the different models, and the ranks of DE genes at FDR 20% between models compared (see Additional file 1: Table S13 and S14).

## Results

### Exploratory analysis

**Fig. 2:**
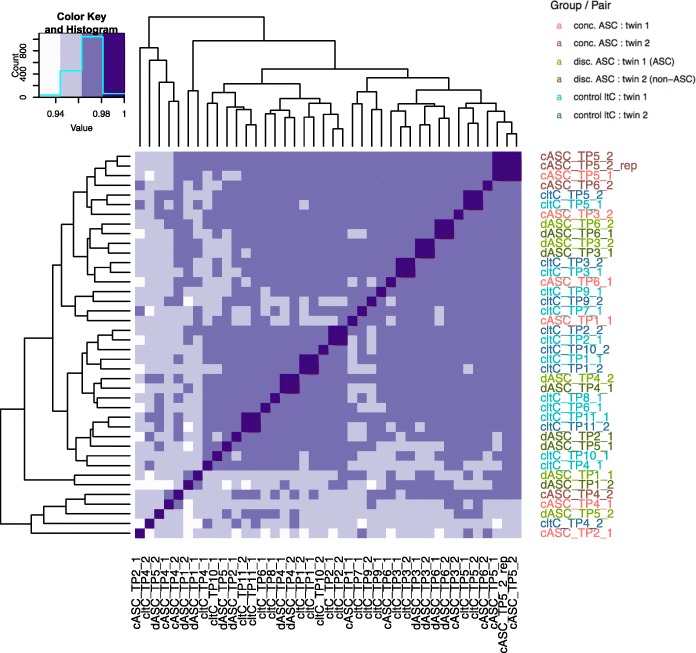
Heatmap showing individuals clustered using correlation of genome-wide expression values between samples as distance (also shown as dendrograms). High correlation *r* values of 0.99 and above are indicated by the dark coloured squares along the diagonal. Twin pairs are found to have an average correlation of 0.98, compared to 0.96 for unrelated subjects. *cASC* concordant ASC, *dASC* discordant ASC, *cltC* concordant low total CAST (control)

Exploratory analysis was performed to assess the quality of the gene expression profiles generated. Gene expression profiles were compared between individuals; high within-twin correlations were observed (median = 0.98), greater than those observed for unrelated individuals (median = 0.96) (Fig. 2), as expected. Next, sample clustering was performed (Additional file 1: Figure S1a,b). The dendrograms showed male and female subjects segregating into two distinct clusters with no sex outliers, and 15 (of the 16) complete twin pairs clustering together in nodes. MDS plots for the first six dimensions were inspected, which showed that samples mainly clustered by sex, case and experimental group, whereas sequencing lane was not seen to segregate the data (Additional file 1: Figure S2). Inspection of these plots also revealed the presence of three outliers, which were subsequently removed from further analysis. Following these steps, 36 samples were taken forward for differential expression analysis (Additional file 1: Table S1a,b and S2a,b).

### Differential expression analysis

**Fig. 3:**
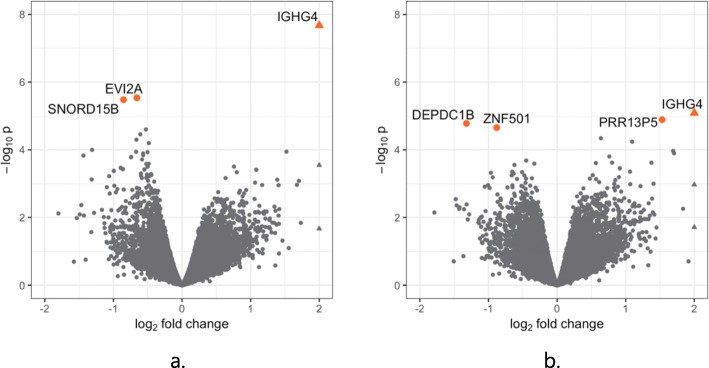
**a**, **b** Volcano plots showing relationship between significance and logFC for **a** discordant and **b** case control analyses. DE genes passing FDR < 0.1 are indicated by the coloured points and labelled

The DE genes identified in the within discordant pairs analysis are presented in Table 1 (with extended list in Additional file 1: Table S3). The volcano plot is shown in Fig. 3a, and examination of the QQ plots is suggestive of uncorrected biases, although we were unable to identify any single factor (see Additional file 1: Figures S3a and S4a). Overall, three genes pass FDR 10%: *IGHG4* (logFC = 2.15, FDR = 0.0004), *EVI2A* (logFC = − 0.66, FDR = 0.02) and *SNORD15B* (logFC = − 0.85, FDR = 0.02). Further down the list of suggestive genes at FDR 20%, a number of ribosomal genes are identified (*RPL9*, *RPL30*, *RPL41*, *RPS3A*). The majority of the top ranked genes were found to have a consistent direction of effect across all discordant pairs (ten out of 16 genes at FDR < 20%), which were all in the direction of decreased expression in ASC affected compared to unaffected co-twins. Additional file 1: Figure S6 shows the expression levels for DE genes at FDR < 10% for each of the twin pairs, stratified by ASC affected status. The direction of the effect is largely consistent for all three genes, with *IGHG4* showing mainly increased expression in ASC cases compared to unaffected co-twin (for four out of five pairs), and *EVI2A* and *SNORD15B* showing decreased expression in all affected twin pair members.

**Table 1:** Discordant group differentially expressed genes at FDR < 20%. For each gene, the estimated fold change, average expression (in log counts per million), *p* value and FDR adjusted *p* values from edgeR are given. The final column indicates if the gene showed a consistent direction of differential expression across all twin pairs and the arrow which direction this was in (increased or decreased expression in ASC affected compared to unaffected co-twin)

ensembl gene id	hgnc symbol	chr	logFC	logCPM	*P* value	FDR	cons. dir
ENSG00000211892	IGHG4	14	2.1483	1.9484	2.11E-08	3.76E-04	
ENSG00000126860	EVI2A	17	− 0.6581	5.1208	2.88E-06	1.94E-02	↓
ENSG00000207445	SNORD15B	11	− 0.8524	3.0638	3.28E-06	1.94E-02	↓
ENSG00000150681	RGS18	1	− 0.5252	6.9877	2.49E-05	1.11E-01	↓
ENSG00000139679	LPAR6	13	− 0.6090	4.7990	3.46E-05	1.23E-01	↓
ENSG00000163682	RPL9	4	− 0.6645	6.8391	5.01E-05	1.48E-01	
ENSG00000163736	PPBP	4	− 0.4830	7.4291	6.32E-05	1.60E-01	↓
ENSG00000035499	DEPDC1B	5	− 1.3113	0.5493	1.00E-04	1.86E-01	↓
ENSG00000187534	PRR13P5	19	1.5220	0.4947	1.14E-04	1.86E-01	
ENSG00000198339	HIST1H4I	6	− 0.6694	3.7498	1.15E-04	1.86E-01	↓
ENSG00000156482	RPL30	8	− 0.5020	8.3615	1.18E-04	1.86E-01	↓
ENSG00000229117	RPL41	12	− 0.5682	6.9233	1.28E-04	1.86E-01	↓
ENSG00000166710	B2M	15	− 0.5655	11.3125	1.44E-04	1.86E-01	↓
ENSG00000138180	CEP55	10	− 1.4366	0.2429	1.48E-04	1.86E-01	
ENSG00000184825	HIST1H2AH	6	− 0.7410	3.6227	1.61E-04	1.86E-01	
ENSG00000145425	RPS3A	4	− 0.7436	6.8823	1.67E-04	1.86E-01

The DE genes from the case-control analysis are presented in Table 2 (extended results in Additional file 1: Table S4), and the volcano plot is shown in Fig. 3b (further plots provided in S3b and S4b). Four genes pass FDR 10%: *IGHG4* (logFC = 2.04, FDR = 0.10), *PRR13P5* (logFC = 1.53, FDR = 0.10), *DEPDC1B* (logFC = − 1.32, FDR = 0.10) and *ZNF501* (logFC = − 0.88, FDR = 0.10), all four of which were also highly ranked in the discordant pairs analysis. Annotation of the 19 genes identified as nominally differentially expressed (FDR < 20%) across the discordant twin and case-control analyses revealed two non-coding RNAs (SNORD15B and AL662889.1), and suggested a bias towards RNA binding activity. See Additional file 1: Table S5.

**Table 2:** Case-control differentially expressed genes at FDR < 20%

Ensembl gene id	HGNC symbol	Chr	logFC	logCPM	*P* value	FDR
ENSG00000211892	IGHG4	14	2.0362	1.0333	8.06E-06	9.73E-02
ENSG00000187534	PRR13P5	19	1.5312	0.3223	1.27E-05	9.73E-02
ENSG00000035499	DEPDC1B	5	− 1.3208	0.4996	1.65E-05	9.73E-02
ENSG00000186446	ZNF501	3	− 0.8795	2.1953	2.19E-05	9.73E-02
ENSG00000224442		2	0.6384	2.9985	4.53E-05	1.61E-01
ENSG00000232184		1	1.0939	1.0182	5.76E-05	1.71E-01

### Within-pair comparisons

To assess whether within-pair expression differences varied between discordant ASC, concordant ASC and concordant low total CAST twins, the distributions of within-pair log FCs were compared (Additional file 1: Figure S5). The mean log FCs differed by group: discordant ASC = 0.44, concordant ASC = 0.39, low total CAST = 0.39 and *t* tests showed this difference was significant when comparing discordant ASC to concordant ASC (*p* < 2.2 × 10^−16^) and discordant ASC to concordant low total CAST (*p* < 2.2 × 10^−16^). The cumulative distributions of the log FCs also differed by group, with KS tests indicating a significant difference between discordant ASC and concordant low total CAST (*p* < 2.2 × 10^−16^), discordant ASC and concordant ASC (8.3 × 10^−14^) and concordant ASC and concordant low total CAST (*p* < 2.2 × 10^−16^). Additional file 1: Figure S7 shows distributions of the expression levels for all four DE genes identified at FDR < 10% in the case-control comparison, stratified by study group and case status. The distribution of expression values can be seen to vary depending on study group, with differences between affected and unaffected co-twins in the discordant ASC appearing on visual inspection to be greater than differences between concordant ASC twin pairs, and concordant low total CAST twin pairs.

### Gene set testing

#### ASC-associated genes

Focused testing of a number of ASC-associated genesets was performed to test for possible enrichment of previously identified ASC-risk genes. The results are presented in Additional file 1: Table S6. There is evidence for enrichment of ASC-associated genes from the SFARI gene set in gene score categories 1 and 2, and categories 3, 4 and 5 in both the discordant and case-control DE gene lists. The iPSYCH-PGC and TADA genesets do not show enrichment in either of the DE gene lists.

### Pathways

The lists of DE genes from both analyses were tested for enrichment of MSigDB pathways. The results are presented in Additional file 1: Tables S7–S10. For the discordant analysis, a number of potentially relevant pathways (unadjusted *p* < 0.05) were identified including those involved in cell signal integration (*mTORC1* signalling), regulation of gene expression (*MYC* targets, *E2F* targets), immune response (Bohn primary immunodeficiency syndrome) and genomic stability (chromosome maintenance). For the case-control analysis, regulation of gene expression (E2F targets, MYC targets), cell signal integration (*mTORC1* signalling, *PI3K AKT mTOR* signalling) and immune response pathways were similarly identified (Browne HCMV infection, Bohn primary immunodeficiency syndrome).

### Integration with DNA methylation data

The gene expression data was integrated with DNA methylation data on the same individuals. The top 20 results are given in Additional file 1: Tables S11 and S12. In the discordant analysis, a number of genes show some combined evidence for differential expression and methylation which is consistent with the findings from DE and pathways analysis implicating immune signalling—*DAPP1*, and regulation of expression/translation: *ZNF501*, *EIF5A2*, *HIST1H2AG.* In the case-control results, *ZNF501*, *DAPP1* and *EIF5A2* were also highly ranked.

### Post hoc sensitivity analyses

Sensitivity analyses were performed, including a male-only analysis and a surrogate variable-based modelling approach intended to capture blood cell composition and other unmeasured batch effects. The identified DE genes were found to be largely robust to the inclusion of surrogate variables in the model, and were not influenced by the exclusion of female samples in the male subjects only analysis (see Additional file 1: Tables S13 and Table S14). In the discordant analysis, the top 3 identified DE genes *IGHG4*, *EVI2A* and *SNORD15B* were consistently found to be within the top 0.03% (rank ≤ 5) to ~ 3% (rank ≤ 600) of DE genes for SV adjusted and male-only analysis. For the case-control analysis, similarly *IGHG4*, *DEPDC1B* and *ZNF501* were also consistently within the top ~ 3%.

## Discussion

### Differential expression

We performed gene expression profiling by RNA-seq in identical twins discordant for ASC. The overall findings are consistent with the hypothesis that gene expression differences in discordant twins could contribute to (or are a consequence of) phenotypic differences, and we further identify specific gene expression differences associated with ASC affected status. In the discordant group analysis comparing gene expression in ASC affected compared to unaffected co-twins, three DE genes were identified at FDR < 10%: *IGHG4*, *EVI2A* and *SNORD15B*. The case control analysis examining expression differences between ASC affected and control samples identified four DE genes at FDR < 10% including *IGHG4*, *DEPDC1B* and *ZNF501.* Comparison of mean within-pair logFC expression differences revealed that differences were greater in the discordant ASC pairs than in concordant ASC and control pairs. Inspection of expression levels for FDR significant genes identified in the case control analysis suggests that larger differences in expression for trait-associated genes are apparent in the discordant ASC pairs.

The top ranked and most highly significant DE gene in both discordant group and case control analyses was *IGHG4*—Immunoglobulin heavy constant gamma 4, which in both analyses showed evidence for upregulation in ASC affected compared to unaffected individuals (discordant: logFC = 2.15, FDR = 0.0004, case control: logFC = 2.04, FDR = 0.0973). This gene codes for an immunoglobin (or antibody) membrane-bound protein produced by B lymphocytes that recognises specific antigens, and upon binding results in immune cell activation. While *IGHG4* does not appear to have previously been linked to ASC, interestingly two previous expression studies using whole blood identified another related and potentially closely interacting immunoglobin *IGHG1* as being upregulated [91, 92] and in one study downregulated [42] in ASC. The existing evidence for the association of *IGHG1* with ASC, together with the observation that immune-related genes and pathways are some of the most common and highly replicated findings in ASC gene expression studies (discussed further below), lends further support to our identification of *IGHG4* as an ASC-associated DE gene.

*DEPDC1B* was the second ranked coding gene in the case-control analysis (logFC = 1.32, FDR = 0.0973). This showed suggestive evidence for association in the discordant analysis (logFC = − 1.3, FDR = 0.186), but is also noteworthy as it showed consistent effect directionality, being downregulated in ASC affected compared to unaffected for all discordant twin pairs. This gene is a member of the DEP domain coding family, and is involved in intracellular signal transduction and cell growth regulation. While it has not previously been associated with ASC, variants in *DEPDC1B* have been associated with intelligence and general cognitive ability in two recent large-scale GWA studies [93, 94]. The evidence taken together suggests that *DEPDC1B* could be an interesting and potentially relevant signal for further follow up study.

Focused testing of ASC-associated gene sets of interest indicated that overall there was enrichment of previously identified ASC loci from SFARI, whilst the iPSYCH-PGC or TADA sets did not show evidence for enrichment. These findings are consistent with those from previous studies that have tested for overlap between established ASC risk loci and ASC DE genes. In a gene mega-analysis of blood-based transcriptomic studies of ASC by Tylee et al. including 626 affected and 447 comparison subjects, the top identified DE genes were not found to be enriched for ASC GWAS associations [18]. Whilst the lack of enrichment for ASC-associated common genetic variants in differentially expressed gene lists might seem surprising, the accumulated evidence would seem to suggest that genetic association and functional approaches are revealing distinct dimensions to the disorder. ASC is frequently characterised as a disorder of the synapse based on genetic findings, whilst in contrast functional findings often implicate other pathways including immune system, inflammation and transcriptional control [95].

Pathway analysis using collections of genes from MSigDB revealed a number of potentially relevant pathways in common between both discordant and case-control analyses. Pathways were identified implicating the immune system (immune cell signalling/signal integration: *mTORC1*, immune response: Browne HCMV infection, Bohn primary immunodeficiency syndrome), transcriptional control (*MYC* targets, *E2F* targets) and chromosome/genomic stability (DNA repair, chromosome maintenance). In support of these findings, a recent comprehensive review of both blood and brain-based transcriptomic studies found that immune system pathways were among the most frequently identified [95]. More specifically, we found replication for the majority of the top pathways: *E2F* targets, *mTOR* and *MYC* pathways identified in a large mega-analysis based on gene expression in blood [18], and immunodeficiency in a meta-analysis using multiple tissues [16].

The integrative analysis revealed a number of genes showing combined evidence for dysregulation in both discordant and case control comparisons that together with the findings from the differential expression and pathways analyses suggest involvement of immune signalling and transcriptional regulation. We highlight *DAPP1* (dual adaptor of phosphotyrosine and 3-phosphoinositides) as a recent large-scale CNV study of > 1600 individuals identified a paternally inherited duplication in two unrelated individuals with ASC that disrupts several exons of *DAPP1* [96], and large duplications and deletions in the gene are reported in the DECHIPER database—the majority of which list autism, autistic behaviours, global developmental delay and/or intellectual disability as the associated phenotype [97].

In the wider context of ASC research, immune system disruption is one of the most consistent findings, with epidemiological studies showing that families with ASC-affected individuals have a higher rate of autoimmune disorders [98, 99], serological studies finding evidence of increased numbers of activated B and NK cells [90] and elevated levels of pro-inflammatory cytokines [100] in peripheral blood samples and post-mortem studies finding evidence of microglial activation in the dorsolateral prefrontal cortex [101]. In addition, there is also a potential causal link between established environmental risk factors such as prenatal viral infection and paternal age, with immune dysregulation and inflammation and increased risk of developmental disorders. In the case of viral infection, animal studies have linked maternal influenza infection to altered brain development and behaviour in mouse models of ASC and schizophrenia [102, 103]. As for older paternal age, it has been suggested that the observed increased incidence of ASC, schizophrenia and bipolar disorder could be the result of immune dysregulation [104] with increases in pro-inflammatory cytokines IL-1β and IL-6 observed in all three disorders [105, 106]. The accumulated evidence from these diverse studies and the potential link to environmental factors makes a compelling case for prioritising the immune-related genes identified here for further study, where we might begin to investigate links to specific environmental exposures. Future studies should be designed in order to establish the molecular drivers, look at the underlying mechanisms, ascertain whether it is primary or secondary to ASC and whether such immune markers might have utility for prognosis, diagnosis or endophenotype classification.

### Strengths and limitations

We used a population-based cohort of 10,000 twins [50] to identify a rare sample of MZ twins discordant for ASC, with the primary aim of identifying biological differences free from genetic confounding. There are a number of drawbacks and limitations with the approach taken. Firstly, as MZ twins stably discordant for ASC are rare, the sample size was constrained. Whilst sample sizes required for 80% power using disease-discordant MZ twins is ~ 15% smaller than case-control designs, based on published EWAS power simulations our N of 40 would not be powered to detect small methylation differences at *p* < 1 × 10^−6^ [107, 108]. While replication using larger sample sizes is required, this study gives an indication of the value of the discordant-twin design and integrative approach for identifying modest biological signals.

Next, there is the use of peripheral blood for expression profiling, since the relationship between individual differences in gene expression in blood and brain is not yet fully understood. Whilst considerable similarities between RNA transcription in blood and brain have been reported for subsets of genes, it is not a prerequisite that peripheral gene expression exactly resembles that of the brain—only that it is a reliable marker of disease and is informative about underlying biological mechanisms [109, 110]. Peripheral blood also offers several important advantages over post-mortem brain tissue such as availability (especially at developmentally relevant time points), control of tissue quality and handling and collection of relevant donor characteristics.

There is the issue of age of the participants at sample collections, since these were collected during adolescence. ASC is a disorder of development and so it is not known whether the molecular differences identified would reflect those present in early development, or whether they are primary or secondary to onset. While the current study is not able to address developmental questions related to onset and trajectory, future genomic studies using prospective birth cohorts containing infants at risk of ASC will be highly valuable in this regard.

One assumption of this study is that that MZ twins are genetically identical. MZ twins have been found to display rare genetic differences, for example point mutations, copy number variants, telomere length, uniparental disomy, somatic mosaicism, chromosomal aneuploidies and mutations in mitochondrial DNA (see [32] for a discussion of these issues). Without comprehensive DNA sequence data on the individuals, the possibility that the observed phenotypic discordances are the result of post-twinning de novo mutations cannot be ruled out. However, this is not likely as whole-genome sequencing and SNP microarray-based studies have failed to find many replicable differences between MZ twins [32, 111], and there have been few reports of discordant MZ twins having a DNA sequence mutation causative for a disease [112].

The integrative analysis performed combining RNA-seq and DNA methylation data on the same individuals was limited in its scope, due to use of low-resolution methylation data from the Illumina 27 K array and absence of genomic data on the same individuals. Therefore, a basic integrative analysis was performed summarising DNA methylation signals at the gene-level, and limiting to identification of *cis* regulatory signals—since the sites covered on the array would be expected to mainly fall in to this category. We opted for evidence in combination and did not perform causal inference, due to the lack of DNA sequence data which is typically used to help establish directionality between the intermediate traits and phenotype. Our top-ranked (FDR < 10%) DE genes do not show evidence of differential methylation in our 27k dataset, but we cannot rule out the role of other *cis* regulatory mechanisms such as histone tail modifications, non-coding RNAs or trans effects. That said, it is encouraging that genes previously associated with ASC showed combined evidence for dysregulation across our gene expression and DNA methylation datasets, and given the limitations of the strategy used, we consider this primarily a useful demonstration of the potential utility of combining a twin-based study design with multi-omics datasets. In future studies, should genotype data and higher resolution methylation data on the same individuals become available, a more comprehensive, genome-wide, site-level eQTM scan could be performed in combination with causal analysis methods to determine the likely relationship between genotype, expression, methylation, and trait [113].

Finally, the current study focused on gene-level expression but the data generated could be utilised to investigate other transcriptomic phenomena such as alternative splicing, allele-specific expression and expression of non-coding RNAs (e.g. lncRNA). Alternative splicing in particular is thought to play a critical role in neuronal development, with some evidence that it could be relevant to ASC pathology [114, 115]. There are also examples of allele-specific gene expression linked to ASC [116, 117].

## Conclusions

We characterised gene expression in a cohort of discordant and concordant ASC twin pairs using blood RNA-seq. The results from the discordant twin analysis revealed enrichment for immune system, transcriptional control and cell cycle regulation pathways in the identified DE genes, which may represent downstream risk pathways where non-shared environmental or stochastic factors converge. We further incorporated DNA methylation profiles on the same individuals and performed a combined analysis to identify putative ASC associated *cis* regulatory signals. While the integrative strategy was limited in its scope, we believe it is a useful demonstration of the potential utility and power of the MZ twins design combined with multi-omics integration for maximising the potential to identify disease-associated signals. We hope the RNA-seq dataset we have generated here will serve as a valuable resource for future investigations into the gene expression patterns underlying ASC (including examination of alternative splicing, allele-specific expression and expression of non-coding RNAs), and work focused on untangling genetic and environmental risk pathways.

## Additional file

Additional file 1: Supplementary results tables and figures. (DOCX 1562 kb)

## Data Availability

Processed read count data can be made available upon request subject to approval by the appropriate ethical boards.
