# Supplementary material for: RNA sequencing of identical twins discordant for autism reveals blood-based signatures implicating immune and transcriptional dysregulation
Source: Mol Autism. 2019 Nov 7;10:38. doi: 10.1186/s13229-019-0285-1 (PMC6839145; doi:10.1186/s13229-019-0285-1)
Supplement: Supplementary file 1 — Supplementary results tables and figures. (DOCX 1562 kb) [file 13229_2019_285_MOESM1_ESM.docx]

|  |  | **sex** | |  |  |  |  | **sex** | |  |
| --- | --- | --- | --- | --- | --- | --- | --- | --- | --- | --- |
|  |  | female | male | **total** |  |  |  | female | male | **total** |
| **case** | ASC | 2 | 11 | 13 |  | **pair status** | pairs (n/2) | 6 | 7 | 26 |
|  | non | 14 | 9 | 23 |  |  | singletons | 4 | 6 | 10 |
|  | **total** | 16 | 20 | 36 |  |  | **total** | 16 | 20 | 36 |

**Table S1 a,b.** Overall sample characteristics following lab and data QC summarised by a. case status and sex, b. by pair status and sex.

|  |  | **sex** | |  |  |  |  | **sex** | |  |
| --- | --- | --- | --- | --- | --- | --- | --- | --- | --- | --- |
|  |  | female | male | **total** |  |  |  | female | male | **total** |
| **case** | ASC | 2 | 3 | 5 |  | **pair status** | pairs (n/2) | 2 | 3 | 10 |
|  | non | 3 | 3 | 6 |  |  | singletons | 1 | 0 | 1 |
|  | **total** | 5 | 6 | 11 |  |  | **total** | 5 | 6 | 11 |

**Table S2 a,b.** Discordant group following lab and data QC summarised by a. case status and sex, b. by pair status and sex.

| **ensembl gene id** | | **hgnc symbol** | **chr** | **logFC** | **logCPM** | **PValue** | **FDR** | **cons. dir** |
| --- | --- | --- | --- | --- | --- | --- | --- | --- |
| ENSG00000211892 | | IGHG4 | 14 | 2.1483 | 1.9484 | 2.11E-08 | 3.76E-04 |  |
| ENSG00000126860 | | EVI2A | 17 | -0.6581 | 5.1208 | 2.88E-06 | 1.94E-02 | ↓ |
| ENSG00000207445 | | SNORD15B | 11 | -0.8524 | 3.0638 | 3.28E-06 | 1.94E-02 | ↓ |
| ENSG00000150681 | | RGS18 | 1 | -0.5252 | 6.9877 | 2.49E-05 | 1.11E-01 | ↓ |
| ENSG00000139679 | | LPAR6 | 13 | -0.6090 | 4.7990 | 3.46E-05 | 1.23E-01 | ↓ |
| ENSG00000163682 | | RPL9 | 4 | -0.6645 | 6.8391 | 5.01E-05 | 1.48E-01 |  |
| ENSG00000163736 | | PPBP | 4 | -0.4830 | 7.4291 | 6.32E-05 | 1.60E-01 | ↓ |
| ENSG00000035499 | | DEPDC1B | 5 | -1.3113 | 0.5493 | 1.00E-04 | 1.86E-01 | ↓ |
| ENSG00000187534 | | PRR13P5 | 19 | 1.5220 | 0.4947 | 1.14E-04 | 1.86E-01 |  |
| ENSG00000198339 | | HIST1H4I | 6 | -0.6694 | 3.7498 | 1.15E-04 | 1.86E-01 | ↓ |
| ENSG00000156482 | | RPL30 | 8 | -0.5020 | 8.3615 | 1.18E-04 | 1.86E-01 | ↓ |
| ENSG00000229117 | | RPL41 | 12 | -0.5682 | 6.9233 | 1.28E-04 | 1.86E-01 | ↓ |
| ENSG00000166710 | | B2M | 15 | -0.5655 | 11.3125 | 1.44E-04 | 1.86E-01 | ↓ |
| ENSG00000138180 | | CEP55 | 10 | -1.4366 | 0.2429 | 1.48E-04 | 1.86E-01 |  |
| ENSG00000184825 | | HIST1H2AH | 6 | -0.7410 | 3.6227 | 1.61E-04 | 1.86E-01 |  |
| ENSG00000145425 | | RPS3A | 4 | -0.7436 | 6.8823 | 1.67E-04 | 1.86E-01 |  |
| ENSG00000122862 | | SRGN | 10 | -0.5821 | 9.3823 | 1.94E-04 | 2.03E-01 |  |
| ENSG00000156508 | | EEF1A1 | 6 | -0.4850 | 11.5574 | 2.60E-04 | 2.45E-01 | ↓ |
| ENSG00000105708 | | ZNF14 | 19 | -0.5202 | 4.2910 | 2.62E-04 | 2.45E-01 | ↓ |
| ENSG00000267436 |  | | 19 | 2.0169 | -0.7101 | 2.87E-04 | 2.55E-01 | ↑ |
| ENSG00000165914 | | TTC7B | 14 | 0.7572 | 2.6127 | 3.16E-04 | 2.57E-01 | ↑ |
| ENSG00000122026 | | RPL21 | 13 | -0.5007 | 7.1253 | 3.19E-04 | 2.57E-01 | ↓ |
| ENSG00000197153 | | HIST1H3J | 6 | -0.8938 | 3.1964 | 3.41E-04 | 2.63E-01 |  |
| ENSG00000186446 | | ZNF501 | 3 | -0.8611 | 2.2176 | 3.71E-04 | 2.65E-01 | ↓ |
| ENSG00000205413 | | SAMD9 | 7 | -0.5474 | 7.3177 | 3.80E-04 | 2.65E-01 |  |
| ENSG00000232184 | | | 1 | 1.0817 | 0.7010 | 3.87E-04 | 2.65E-01 | ↑ |
| ENSG00000100890 | | KIAA0391 | 14 | -0.9949 | 0.8107 | 4.19E-04 | 2.76E-01 | ↓ |
| ENSG00000127415 | | IDUA | 4 | 0.7981 | 2.3560 | 4.57E-04 | 2.90E-01 | ↑ |
| ENSG00000163221 | | S100A12 | 1 | -0.5982 | 5.3608 | 4.93E-04 | 3.02E-01 |  |
| ENSG00000143401 | | ANP32E | 1 | -0.4503 | 5.0116 | 5.14E-04 | 3.04E-01 | ↓ |
| ENSG00000155304 | | HSPA13 | 21 | -0.5102 | 5.1134 | 5.30E-04 | 3.04E-01 |  |
| ENSG00000134419 | | RPS15A | 16 | -0.4460 | 6.1990 | 5.51E-04 | 3.06E-01 | ↓ |
| ENSG00000168242 | | HIST1H2BI | 6 | -0.5984 | 4.4622 | 5.75E-04 | 3.06E-01 |  |
| ENSG00000200312 | | RN7SKP255 | 14 | -1.0622 | 3.3652 | 5.95E-04 | 3.06E-01 | ↓ |
| ENSG00000127920 | | GNG11 | 7 | -0.4787 | 4.5212 | 6.03E-04 | 3.06E-01 | ↓ |
| ENSG00000177888 | | ZBTB41 | 1 | -0.5191 | 5.3229 | 6.62E-04 | 3.18E-01 |  |
| ENSG00000224442 | | | 2 | 0.6348 | 2.9032 | 6.79E-04 | 3.18E-01 | ↑ |
| ENSG00000075239 | | ACAT1 | 11 | -0.5836 | 3.3051 | 6.79E-04 | 3.18E-01 | ↓ |
| ENSG00000262202 | | SNORD3D | 17 | -1.3154 | 0.0443 | 7.47E-04 | 3.38E-01 | ↓ |
| ENSG00000236029 | | | 9 | 1.3876 | 0.4938 | 7.60E-04 | 3.38E-01 | ↑ |
| ENSG00000120526 | | NUDCD1 | 8 | -0.4955 | 4.0878 | 8.15E-04 | 3.48E-01 | ↓ |
| ENSG00000002726 | | AOC1 | 7 | 1.7039 | 0.1037 | 8.23E-04 | 3.48E-01 | ↑ |
| ENSG00000231500 | | RPS18 | 6 | -0.4052 | 8.1880 | 8.67E-04 | 3.58E-01 | ↓ |
| ENSG00000257390 | | | 12 | 1.0637 | 0.4641 | 9.28E-04 | 3.75E-01 | ↑ |
| ENSG00000138395 | | CDK15 | 2 | 1.6745 | -0.0244 | 1.08E-03 | 4.14E-01 |  |
| ENSG00000235316 | | DUSP8P5 | 10 | 1.1685 | 0.2696 | 1.09E-03 | 4.14E-01 | ↑ |
| ENSG00000262652 | | | 17 | 1.4062 | 0.1977 | 1.11E-03 | 4.14E-01 |  |
| ENSG00000196787 | | HIST1H2AG | 6 | -0.4836 | 5.0695 | 1.12E-03 | 4.14E-01 |  |
| ENSG00000200959 | | SNORA74A | 5 | -0.9872 | 1.9440 | 1.15E-03 | 4.17E-01 |  |

**Table S3**. Discordant group top 50 DE genes (by p value). For each gene, the estimated fold change, average expression (in log counts per million), p value, and FDR adusted p values from edgeR are given. The final column indicates if the gene showed a consistent direction of differential expression across all twin pairs and the arrow which direction this was in (increased or decreased expression in ASC affected compared to unaffected cotwin). DE genes above the red line are those passing FDR 20%.

| **ensembl gene id** | **hgnc symbol** | **chr** | **logFC** | **logCPM** | **PValue** | **FDR** |
| --- | --- | --- | --- | --- | --- | --- |
| ENSG00000211892 | IGHG4 | 14 | 2.0362 | 1.0333 | 8.06E-06 | 9.73E-02 |
| ENSG00000187534 | PRR13P5 | 19 | 1.5312 | 0.3223 | 1.27E-05 | 9.73E-02 |
| ENSG00000035499 | DEPDC1B | 5 | -1.3208 | 0.4996 | 1.65E-05 | 9.73E-02 |
| ENSG00000186446 | ZNF501 | 3 | -0.8795 | 2.1953 | 2.19E-05 | 9.73E-02 |
| ENSG00000224442 | AC017035.1 | 2 | 0.6384 | 2.9985 | 4.53E-05 | 1.61E-01 |
| ENSG00000232184 | AL662889.1 | 1 | 1.0939 | 1.0182 | 5.76E-05 | 1.71E-01 |
| ENSG00000138395 | CDK15 | 2 | 1.6917 | 0.6773 | 1.08E-04 | 2.73E-01 |
| ENSG00000002726 | AOC1 | 7 | 1.7046 | 0.4519 | 1.26E-04 | 2.80E-01 |
| ENSG00000165914 | TTC7B | 14 | 0.7613 | 2.7996 | 1.58E-04 | 3.11E-01 |
| ENSG00000143401 | ANP32E | 1 | -0.4499 | 4.8343 | 2.08E-04 | 3.27E-01 |
| ENSG00000127415 | IDUA | 4 | 0.8017 | 2.3888 | 2.41E-04 | 3.27E-01 |
| ENSG00000236029 | | 9 | 1.3750 | 0.3559 | 2.41E-04 | 3.27E-01 |
| ENSG00000205930 | C21orf49 | 21 | 0.5654 | 2.2381 | 2.47E-04 | 3.27E-01 |
| ENSG00000185880 | TRIM69 | 15 | -0.3315 | 5.8279 | 2.58E-04 | 3.27E-01 |
| ENSG00000179271 | GADD45GIP1 | 19 | -0.6220 | 2.8306 | 2.81E-04 | 3.32E-01 |
| ENSG00000260872 | | 16 | 0.9142 | 1.6349 | 3.51E-04 | 3.90E-01 |
| ENSG00000257390 | | 12 | 1.0623 | 0.4826 | 4.39E-04 | 4.19E-01 |
| ENSG00000155755 | TMEM237 | 2 | -0.6542 | 1.7881 | 4.41E-04 | 4.19E-01 |
| ENSG00000155304 | HSPA13 | 21 | -0.5101 | 4.9719 | 4.52E-04 | 4.19E-01 |
| ENSG00000264575 | LINC00526 | 18 | -0.9653 | 0.6281 | 4.73E-04 | 4.19E-01 |
| ENSG00000153485 | TMEM251 | 14 | -0.6332 | 2.1719 | 4.99E-04 | 4.19E-01 |
| ENSG00000101104 | PABPC1L | 20 | 0.5204 | 3.6252 | 5.57E-04 | 4.19E-01 |
| ENSG00000198339 | HIST1H4I | 6 | -0.6612 | 3.6439 | 5.64E-04 | 4.19E-01 |
| ENSG00000136514 | RTP4 | 3 | -0.4752 | 3.6000 | 5.68E-04 | 4.19E-01 |
| ENSG00000188282 | RUFY4 | 2 | 0.9017 | 0.9365 | 6.36E-04 | 4.19E-01 |
| ENSG00000205544 | TMEM256 | 17 | -0.9378 | 1.2279 | 6.46E-04 | 4.19E-01 |
| ENSG00000065911 | MTHFD2 | 2 | -0.3670 | 4.5810 | 6.87E-04 | 4.19E-01 |
| ENSG00000165629 | ATP5C1 | 10 | -0.4523 | 5.1875 | 7.05E-04 | 4.19E-01 |
| ENSG00000144589 | STK11IP | 2 | 0.4785 | 3.7052 | 7.06E-04 | 4.19E-01 |
| ENSG00000075239 | ACAT1 | 11 | -0.5833 | 3.0784 | 7.21E-04 | 4.19E-01 |
| ENSG00000214226 | C17orf67 | 17 | 0.5693 | 2.3047 | 7.30E-04 | 4.19E-01 |
| ENSG00000174945 | AMZ1 | 7 | 0.8483 | 0.7207 | 7.79E-04 | 4.32E-01 |
| ENSG00000273151 | | 7 | 0.6141 | 2.7773 | 8.98E-04 | 4.58E-01 |
| ENSG00000136122 | BORA | 13 | -0.4597 | 3.2548 | 9.41E-04 | 4.58E-01 |
| ENSG00000262652 | | 17 | 1.4045 | 0.2541 | 1.00E-03 | 4.58E-01 |
| ENSG00000234737 | KRT18P15 | 3 | 0.8346 | 1.1851 | 1.01E-03 | 4.58E-01 |
| ENSG00000239697 | TNFSF12 | 17 | 1.0663 | 0.9076 | 1.03E-03 | 4.58E-01 |
| ENSG00000267436 | | 19 | 2.0007 | -0.5249 | 1.07E-03 | 4.58E-01 |
| ENSG00000260339 | HEXA-AS1 | 15 | -1.0060 | 0.6518 | 1.10E-03 | 4.58E-01 |
| ENSG00000260093 | | 8 | -0.6515 | 1.8210 | 1.11E-03 | 4.58E-01 |
| ENSG00000198843 | | 3 | -0.3911 | 6.3643 | 1.14E-03 | 4.58E-01 |
| ENSG00000235162 | C12orf75 | 12 | -0.5177 | 3.5729 | 1.16E-03 | 4.58E-01 |
| ENSG00000265416 | | 2 | 1.2156 | 0.3950 | 1.18E-03 | 4.58E-01 |
| ENSG00000182004 | SNRPE | 1 | -0.4996 | 3.1385 | 1.21E-03 | 4.58E-01 |
| ENSG00000176945 | MUC20 | 3 | 0.8864 | 0.8964 | 1.22E-03 | 4.58E-01 |
| ENSG00000207137 | SNORD116-13 | 15 | -1.0543 | 0.2706 | 1.23E-03 | 4.58E-01 |
| ENSG00000273148 | | 20 | -0.6493 | 1.7404 | 1.26E-03 | 4.58E-01 |
| ENSG00000103260 | METRN | 16 | -0.7222 | 1.0989 | 1.29E-03 | 4.58E-01 |
| ENSG00000086598 | TMED2 | 12 | -0.3144 | 6.5502 | 1.31E-03 | 4.58E-01 |

**Table S4.** Case-control Top 50 DE genes (by p value). For each gene, the estimated fold change, average expression (in log counts per million), p value, and FDR adjusted p values from edgeR are given. The final column indicates if the gene showed a consistent direction of differential expression across all twin pairs and the arrow which direction this was in (increased or decreased expression in ASC affected compared to unaffected cotwin). DE genes above the red line are those passing FDR 20%.

| **ensembl gene id** | **Gene name** | **Gene type** | **chr** | **GO molecular function** | **comment** |
| --- | --- | --- | --- | --- | --- |
| ENSG00000035499 | DEPDC1B | protein coding | chr5 | GTPase activator activity | -- |
| ENSG00000126860 | EVI2A | protein coding | chr17 | transmembrane signalling receptor activity | overlapping locus: exon(s) overlap exon(s) of a readthrough transcript or a transcript belonging to another locus |
| ENSG00000138180 | CEP55 | protein coding | chr10 | protein binding | -- |
| ENSG00000139679 | LPAR6 | protein coding | chr13 | G protein-coupled receptor activity | -- |
| ENSG00000145425 | RPS3A | protein coding | chr4 | RNA binding; structural constituent of ribosome; protein binding; mRNA 5'-UTR binding | ncRNA_host: locus is a host for small non-coding RNAs |
| ENSG00000150681 | RGS18 | protein coding | chr1 | GTPase activity; GTPase activator activity; protein binding | -- |
| ENSG00000156482 | RPL30 | protein coding | chr8 | RNA binding; structural constituent of ribosome; protein binding; selenocysteine insertion sequence binding | ncRNA_host |
| ENSG00000163682 | RPL9 | protein coding | chr4 | RNA binding; structural constituent of ribosome; protein binding; rRNA binding | -- |
| ENSG00000163736 | PPBP | protein coding | chr4 | cytokine activity; glucose transmembrane transporter activity; protein binding; chemokine activity; growth factor activity | -- |
| ENSG00000166710 | B2M | protein coding | chr15 | protein binding | -- |
| ENSG00000184825 |  | -- |  |  | -- |
| ENSG00000186446 | ZNF501 | protein coding | chr3 | DNA-binding transcription factor activity, RNA polymerase II-specific; nucleic acid binding; DNA binding; DNA-binding transcription factor activity; protein binding | -- |
| ENSG00000187534 | PRR13P5 | processed pseudogene | chr19 | -- | member of the pseudogene set predicted by YALE, UCSC and HAVANA |
| ENSG00000198339 |  | -- |  | -- | -- |
| ENSG00000207445 | SNORD15B | snoRNA | chr11 | predicated to guide the 2'O-ribose methylation of 28S rRNA A3764 | -- |
| ENSG00000211892 | IGHG4 | IG_C_gene | chr14 | antigen binding; immunoglobulin receptor binding | -- |
| ENSG00000224442 | AC017035.1 | Processed pseudogene | chr2 | -- | member of the pseudogene set ; overlapping locus |
| ENSG00000229117 | RPL41 | protein coding | chr12 | RNA binding; mRNA 3'-UTR binding; structural constituent of ribosome; protein binding; mRNA 5'-UTR binding | -- |
| ENSG00000232184 | AL662889.1 | lncRNA | chr1 | -- | overlapping locus |

**Table S5.** Classification of gene features for all DE genes (FDR <20%) identified in both MZ twin and case-control analyses. Annotation based on GENCODE (release 31) and GO terms. Processed pseudogene = Pseudogene that lack introns and is thought to arise from reverse transcription of mRNA followed by reinsertion of DNA into the genome; snoRNA = small nucleolar RNA; lncRNA = long non-coding RNA; IG_C_gene = functional Immunoglobulin (Ig) variable chain gene

|  | **geneset enrichment p** | | | |
| --- | --- | --- | --- | --- |
|  | SFARI | SFARI | IPSYCH-PGC | TADA |
| **DE gene list** | GS 1,2 | GS 3,4,5 |  |  |
| discordant group | 0.01 | 0.01 | 0.22 | 0.19 |
| case control | 0.002 | 0.003 | 0.16 | 0.07 |

**Table S6.** Enrichment analysis p-values for ASC related genesets for both discordant group and case control results lists.

| **pathway** | **N** | **p-value** | **FDR** |
| --- | --- | --- | --- |
| HALLMARK_MYC_TARGETS_V1 | 199 | 0.037 | 0.817 |
| HALLMARK_MTORC1_SIGNALING | 196 | 0.042 | 0.817 |
| HALLMARK_E2F_TARGETS | 196 | 0.05 | 0.817 |
| HALLMARK_G2M_CHECKPOINT | 191 | 0.09 | 0.999 |
| HALLMARK_OXIDATIVE_PHOSPHORYLATION | 200 | 0.134 | 0.999 |
| HALLMARK_UNFOLDED_PROTEIN_RESPONSE | 107 | 0.207 | 0.999 |
| HALLMARK_MITOTIC_SPINDLE | 194 | 0.213 | 0.999 |
| HALLMARK_SPERMATOGENESIS | 71 | 0.219 | 0.999 |
| HALLMARK_PROTEIN_SECRETION | 90 | 0.232 | 0.999 |
| HALLMARK_MYC_TARGETS_V2 | 58 | 0.276 | 0.999 |
| HALLMARK_ADIPOGENESIS | 176 | 0.301 | 0.999 |
| HALLMARK_ANDROGEN_RESPONSE | 86 | 0.301 | 0.999 |
| HALLMARK_KRAS_SIGNALING_UP | 143 | 0.304 | 0.999 |
| HALLMARK_DNA_REPAIR | 145 | 0.371 | 0.999 |
| HALLMARK_APOPTOSIS | 141 | 0.371 | 0.999 |
| HALLMARK_REACTIVE_OXIGEN_SPECIES_PATHWAY | 49 | 0.424 | 0.999 |
| HALLMARK_NOTCH_SIGNALING | 27 | 0.437 | 0.999 |
| HALLMARK_ALLOGRAFT_REJECTION | 168 | 0.517 | 0.999 |
| HALLMARK_PEROXISOME | 88 | 0.532 | 0.999 |
| HALLMARK_UV_RESPONSE_DN | 113 | 0.537 | 0.999 |

**Table S7**. Discordant group top hallmark pathways. For each pathway, the size of the geneset is given along with the p-value for the mixed hypothesis testing if genes in the set tend to be up or down regulated, and the FDR adjusted p-value.

| **pathway** | **N** | **p-value** | **FDR** |
| --- | --- | --- | --- |
| WONG_EMBRYONIC_STEM_CELL_CORE | 319 | 0.001 | 0.410 |
| SASAKI_ADULT_T_CELL_LEUKEMIA | 160 | 0.001 | 0.410 |
| SAKAI_TUMOR_INFILTRATING_MONOCYTES_DN | 72 | 0.001 | 0.410 |
| RHODES_UNDIFFERENTIATED_CANCER | 65 | 0.001 | 0.41 |
| BOHN_PRIMARY_IMMUNODEFICIENCY_SYNDROM_UP | 45 | 0.001 | 0.41 |
| FOURNIER_ACINAR_DEVELOPMENT_LATE_DN | 19 | 0.001 | 0.41 |
| NIKOLSKY_BREAST_CANCER_15Q26_AMPLICON | 19 | 0.001 | 0.41 |
| REACTOME_SIGNALING_BY_CONSTITUTIVELY_ACTIVE_EGFR | 16 | 0.001 | 0.41 |
| LEE_LIVER_CANCER_SURVIVAL_DN | 162 | 0.002 | 0.504 |
| REACTOME_CHROMOSOME_MAINTENANCE | 87 | 0.002 | 0.504 |
| ZHANG_RESPONSE_TO_CANTHARIDIN_DN | 66 | 0.002 | 0.504 |
| REACTOME_DEPOSITION_OF_NEW_CENPA_CONTAINING… | 37 | 0.002 | 0.504 |
| LIANG_HEMATOPOIESIS_STEM_CELL_NUMBER_SMALL… | 36 | 0.002 | 0.504 |
| WEST_ADRENOCORTICAL_TUMOR_UP | 274 | 0.003 | 0.615 |
| ACEVEDO_NORMAL_TISSUE_ADJACENT_TO_LIVER_TUMOR_UP | 146 | 0.003 | 0.615 |
| HUTTMANN_B_CLL_POOR_SURVIVAL_DN | 58 | 0.003 | 0.615 |
| RHEIN_ALL_GLUCOCORTICOID_THERAPY_DN | 346 | 0.004 | 0.713 |
| BIOCARTA_DEATH_PATHWAY | 32 | 0.004 | 0.713 |
| BROWNE_HCMV_INFECTION_30MIN_DN | 106 | 0.005 | 0.713 |
| BHATTACHARYA_EMBRYONIC_STEM_CELL | 72 | 0.005 | 0.713 |

**Table S8.** discordant group top curated pathways. For each pathway, the size of the geneset is given along with the p-value for the mixed hypothesis testing if genes in the set tend to be up or down regulated, and the FDR adjusted p-value.

| **pathway** | **N** | **p-value** | **FDR** |
| --- | --- | --- | --- |
| HALLMARK_E2F_TARGETS | 196 | 0.023 | 0.882 |
| HALLMARK_MYC_TARGETS_V1 | 199 | 0.047 | 0.882 |
| HALLMARK_MTORC1_SIGNALING | 196 | 0.062 | 0.882 |
| HALLMARK_OXIDATIVE_PHOSPHORYLATION | 200 | 0.085 | 0.882 |
| HALLMARK_ANDROGEN_RESPONSE | 86 | 0.09 | 0.882 |
| HALLMARK_G2M_CHECKPOINT | 191 | 0.114 | 0.907 |
| HALLMARK_MITOTIC_SPINDLE | 194 | 0.134 | 0.907 |
| HALLMARK_MYC_TARGETS_V2 | 58 | 0.176 | 0.907 |
| HALLMARK_UNFOLDED_PROTEIN_RESPONSE | 107 | 0.18 | 0.907 |
| HALLMARK_PROTEIN_SECRETION | 90 | 0.185 | 0.907 |
| HALLMARK_NOTCH_SIGNALING | 27 | 0.239 | 0.96 |
| HALLMARK_APOPTOSIS | 141 | 0.242 | 0.96 |
| HALLMARK_SPERMATOGENESIS | 71 | 0.276 | 0.96 |
| HALLMARK_ADIPOGENESIS | 176 | 0.292 | 0.96 |
| HALLMARK_ALLOGRAFT_REJECTION | 168 | 0.323 | 0.96 |
| HALLMARK_TGF_BETA_SIGNALING | 49 | 0.328 | 0.96 |
| HALLMARK_UV_RESPONSE_DN | 113 | 0.333 | 0.96 |
| HALLMARK_PI3K_AKT_MTOR_SIGNALING | 91 | 0.371 | 0.997 |
| HALLMARK_DNA_REPAIR | 145 | 0.399 | 0.997 |
| HALLMARK_KRAS_SIGNALING_UP | 143 | 0.444 | 0.997 |

**Table S9.** case-control top hallmark pathways. For each pathway, the size of the geneset is given along with the p-value for the mixed hypothesis testing if genes in the set tend to be up or down regulated, and the FDR adjusted p-value.

| **pathway** | **N** | **p-value** | **FDR** |
| --- | --- | --- | --- |
| BROWNE_HCMV_INFECTION_30MIN_DN | 106 | 0.001 | 0.62 |
| BOHN_PRIMARY_IMMUNODEFICIENCY_SYNDROM_UP | 45 | 0.001 | 0.62 |
| FUJII_YBX1_TARGETS_UP | 28 | 0.001 | 0.62 |
| CHIARETTI_T_ALL_RELAPSE_PROGNOSIS | 19 | 0.001 | 0.62 |
| HORIUCHI_WTAP_TARGETS_DN | 278 | 0.002 | 0.62 |
| REACTOME_MEIOSIS | 65 | 0.002 | 0.62 |
| BOYLAN_MULTIPLE_MYELOMA_D_CLUSTER_UP | 23 | 0.002 | 0.62 |
| WANG_RESPONSE_TO_FORSKOLIN_UP | 18 | 0.002 | 0.62 |
| MA_PITUITARY_FETAL_VS_ADULT_DN | 16 | 0.002 | 0.62 |
| LEE_LIVER_CANCER_SURVIVAL_DN | 162 | 0.003 | 0.62 |
| GARCIA_TARGETS_OF_FLI1_AND_DAX1_DN | 149 | 0.003 | 0.62 |
| ACEVEDO_LIVER_CANCER_WITH_H3K27ME3_DN | 106 | 0.003 | 0.62 |
| BHATTACHARYA_EMBRYONIC_STEM_CELL | 72 | 0.003 | 0.62 |
| BIOCARTA_AKT_PATHWAY | 19 | 0.003 | 0.62 |
| LINDGREN_BLADDER_CANCER_CLUSTER_3_UP | 288 | 0.004 | 0.62 |
| KOKKINAKIS_METHIONINE_DEPRIVATION_96HR_UP | 96 | 0.004 | 0.62 |
| WONG_IFNA2_RESISTANCE_DN | 26 | 0.004 | 0.62 |
| BIOCARTA_IGF1MTOR_PATHWAY | 19 | 0.004 | 0.62 |
| NIKOLSKY_BREAST_CANCER_15Q26_AMPLICON | 19 | 0.004 | 0.62 |
| CHOI_ATL_CHRONIC_VS_ACUTE_DN | 18 | 0.004 | 0.62 |

**Table S10.** case-control top curated pathways. For each pathway, the size of the geneset is given along with the p-value for the mixed hypothesis testing if genes in the set tend to be up or down regulated, and the FDR adjusted p-value.

| **gene** | **P value 27K** | **P value RNA seq** | **combined P value** | **cross-platform correlation** |
| --- | --- | --- | --- | --- |
| CEP55 | 1.13E-01 | 1.48E-04 | 2.01E-04 | 0.0552 |
| DNAJA1 | 2.33E-02 | 1.74E-03 | 4.51E-04 | -0.1016 |
| RPS3A | 2.56E-01 | 1.67E-04 | 5.61E-04 | 0.0707 |
| ANXA1 | 3.66E-04 | 1.55E-01 | 9.35E-04 | 0.3060 |
| DAPP1 | 1.69E-03 | 7.21E-02 | 1.32E-03 | -0.1930 |
| HIST1H2AG | 1.47E-01 | 1.12E-03 | 1.59E-03 | -0.2611 |
| ACN9 | 2.80E-03 | 3.21E-02 | 1.69E-03 | 0.2281 |
| RPS15A | 3.34E-01 | 5.51E-04 | 1.77E-03 | -0.3277 |
| MOCS3 | 1.68E-02 | 1.20E-02 | 1.92E-03 | -0.1688 |
| ZNF501 | 5.68E-01 | 3.71E-04 | 1.99E-03 | 0.1120 |
| ZNRF1 | 2.30E-02 | 9.27E-03 | 2.01E-03 | -0.0582 |
| ALKBH6 | 4.32E-02 | 6.15E-03 | 2.45E-03 | -0.0934 |
| IDUA | 6.04E-01 | 4.57E-04 | 2.53E-03 | -0.0995 |
| EIF5A2 | 2.32E-02 | 8.28E-03 | 3.14E-03 | 0.1001 |
| MMP8 | 2.65E-02 | 1.00E-02 | 3.21E-03 | -0.0408 |
| TAS2R60 | 6.96E-03 | 2.57E-02 | 3.42E-03 | 0.0063 |
| FCRL1 | 2.03E-02 | 2.14E-02 | 3.79E-03 | 0.2104 |
| CAPZA1 | 1.65E-01 | 1.88E-03 | 3.87E-03 | 0.1591 |
| C11orf49 | 1.20E-02 | 3.85E-02 | 4.00E-03 | -0.2284 |
| ATP9A | 1.36E-03 | 3.60E-01 | 4.21E-03 | 0.0002 |

**Table S11.** Discordant group top 20 integration results. P values indicating the evidence for differential methylation (from the 27K methylation dataset) and differential expression (as assessed in the current study) are given along with the combined p value. Cross platform correlation gives the level of concordance between the gene-level DNA methylation and gene expression measurements, with high values taken as indicative of *cis* regulation. Results are sorted by combined p value.

| **gene** | **P value 27K** | **P value RNA seq** | **combined P value** | **cross-platform correlation** |
| --- | --- | --- | --- | --- |
| ZNF501 | 5.56E-01 | 2.19E-05 | 1.99E-04 | 0.1120 |
| DAPP1 | 1.64E-03 | 4.17E-02 | 7.27E-04 | -0.1930 |
| ZNRF1 | 1.68E-02 | 4.51E-03 | 7.96E-04 | -0.0582 |
| SNRPE | 7.87E-02 | 1.21E-03 | 9.79E-04 | -0.1117 |
| MOCS3 | 2.96E-02 | 4.37E-03 | 1.29E-03 | -0.1688 |
| NOV | 9.21E-03 | 1.64E-02 | 1.48E-03 | -0.0246 |
| DNAJA1 | 1.44E-02 | 1.34E-02 | 1.84E-03 | -0.1016 |
| TMED2 | 1.64E-01 | 1.31E-03 | 2.03E-03 | 0.0298 |
| PFKP | 2.50E-04 | 8.76E-01 | 2.06E-03 | -0.2794 |
| PARP6 | 6.31E-02 | 3.41E-03 | 2.26E-03 | 0.1774 |
| AMPD3 | 8.29E-03 | 2.15E-02 | 2.32E-03 | -0.1438 |
| KIAA0895 | 4.62E-03 | 4.38E-02 | 2.34E-03 | 0.1786 |
| EIF5A2 | 3.28E-02 | 6.40E-03 | 2.70E-03 | 0.1001 |
| TAS2R60 | 7.70E-03 | 2.38E-02 | 2.72E-03 | 0.0063 |
| RECQL5 | 1.76E-03 | 1.99E-01 | 3.16E-03 | 0.0160 |
| ASF1A | 2.72E-02 | 1.41E-02 | 3.39E-03 | -0.2996 |
| SPATA6 | 1.59E-01 | 2.62E-03 | 3.65E-03 | -0.0423 |
| NBN | 2.96E-02 | 1.02E-02 | 3.75E-03 | 0.1380 |
| SSFA2 | 3.58E-03 | 8.10E-02 | 3.86E-03 | 0.2712 |
| FCRL1 | 3.91E-02 | 8.21E-03 | 3.95E-03 | 0.2104 |

**Table S12.** Case-control top 20 integration results. P values indicating the evidence for differential methylation (from the 27K methylation dataset) and differential expression (as assessed in the current study) are given along with the combined p value. Cross platform correlation gives the level of concordance between the gene-level DNA methylation and gene expression measurements, with high values taken as indicative of *cis* regulation. Results are sorted by combined p value.

|  |  | **discordant ASD** | | **case-control** | | **discordant ASD SV model** | | **discordant ASD males** | | **discordant ASD males SV model** | |
| --- | --- | --- | --- | --- | --- | --- | --- | --- | --- | --- | --- |
| **ensembl_gene_id** | **hgnc_symbol** | **logFC** | **rank** | **logFC** | **rank** | **logFC** | **rank** | **logFC** | **rank** | **logFC** | **rank** |
| ENSG00000211892 | IGHG4 | 2.1483 | 1 | 2.0362 | 1 | 4.2112 | 5 | 1.8358 | 6 | 2.1340 | 52 |
| ENSG00000126860 | EVI2A | -0.6581 | 2 | -0.6556 | 188 | -0.5762 | 173 | -0.5866 | 78 | -0.4829 | 91 |
| ENSG00000207445 | SNORD15B | -0.8524 | 3 | -0.8254 | 409 | -0.8994 | 564 | -0.8749 | 28 | -0.6992 | 578 |
| ENSG00000150681 | RGS18 | -0.5252 | 4 | -0.5264 | 58 | -0.3765 | 570 | -0.3827 | 414 | -0.3102 | 617 |
| ENSG00000139679 | LPAR6 | -0.6090 | 5 | -0.6242 | 65 | -0.2879 | 2496 | -0.5382 | 130 | -0.4462 | 190 |
| ENSG00000163682 | RPL9 | -0.6645 | 6 | -0.6663 | 469 | -0.7967 | 19 | -0.6565 | 149 | -0.7158 | 61 |
| ENSG00000163736 | PPBP | -0.4830 | 7 | -0.4831 | 139 | -0.4212 | 298 | -0.4760 | 187 | -0.4266 | 293 |
| ENSG00000035499 | DEPDC1B | -1.3113 | 8 | -1.3208 | 3 | -0.2232 | 13348 | -1.3337 | 148 | -0.5971 | 4896 |
| ENSG00000187534 | PRR13P5 | 1.5220 | 9 | 1.5312 | 2 | 2.8751 | 1217 | 1.6569 | 8 | 1.5202 | 607 |
| ENSG00000198339 | HIST1H4I | -0.6694 | 10 | -0.6612 | 23 | -0.7069 | 108 | -0.7088 | 58 | -0.5422 | 214 |
| ENSG00000156482 | RPL30 | -0.5020 | 11 | -0.5021 | 174 | -0.3323 | 859 | -0.5320 | 138 | -0.4196 | 555 |
| ENSG00000229117 | RPL41 | -0.5682 | 12 | -0.5698 | 445 | -0.4899 | 109 | -0.6260 | 86 | -0.5733 | 113 |
| ENSG00000166710 | B2M | -0.5655 | 13 | -0.5655 | 272 | -0.4776 | 486 | -0.5224 | 409 | -0.4426 | 1200 |
| ENSG00000138180 | CEP55 | -1.4366 | 14 | -1.3560 | 221 | -0.0390 | 17342 | -1.3228 | 398 | -0.2740 | 12443 |
| ENSG00000184825 | HIST1H2AH | -0.7410 | 15 | -0.7101 | 482 | -0.4801 | 1228 | -0.7407 | 143 | -0.4440 | 1206 |
| ENSG00000145425 | RPS3A | -0.7436 | 16 | -0.7461 | 658 | -0.9394 | 14 | -0.7049 | 335 | -0.8515 | 66 |

**Table S13.** Discordant group sensitivity analysis. Differential expression analyses were re-run for the discordant group using males-only (N= 6) and using a surrogate variable (SV) analysis that infers cell counts in order to adjust for (unmeasured) cellular heterogeneity. LogFC and rank of the differentially expressed genes (FDR < 20%) identified in the main discordant twin analysis are reported following a) SV adjustment b) exclusion of female samples and c) male-only and SV analysis combined.

|  | |  | **case control** | | **case-control SV model** | | **case-control males** | | **case-control males SV model** | |
| --- | --- | --- | --- | --- | --- | --- | --- | --- | --- | --- |
| **ensembl_gene_id** | | **hgnc_symbol** | **logFC** | **rank** | **logFC** | **rank** | **logFC** | **rank** | **logFC** | **rank** |
| ENSG00000211892 | | IGHG4 | 2.0362 | 1 | 1.7927 | 66 | 1.8173 | 3 | 2.2484 | 16 |
| ENSG00000187534 | PRR13P5 | | 1.5312 | 2 | 1.2739 | 580 | 1.6671 | 20 | 1.9560 | 325 |
| ENSG00000035499 | | DEPDC1B | -1.3208 | 3 | -1.0057 | 229 | -1.3726 | 62 | -0.8162 | 1758 |
| ENSG00000186446 | | ZNF501 | -0.8795 | 4 | -0.5239 | 438 | -1.0724 | 8 | -0.6717 | 194 |
| ENSG00000224442 | | | 0.6384 | 5 | 0.5978 | 2 | 0.4486 | 1405 | 0.5760 | 135 |
| ENSG00000232184 | | | 1.0939 | 6 | 0.9037 | 98 | 0.8398 | 637 | 0.7783 | 1125 |

**Table S14.** Case-control sensitivity analysis. Differential expression analyses were re-run for the case-control comparison using males-only (N=20, 11 ASC cases ) and using a surrogate variable (SV) analysis that infers cell counts in order to adjust for (unmeasured) cellular heterogeneity. LogFC and rank of the differentially expressed genes (FDR < 20%) identified in the main case-control analysis are reported following a) SV adjustment b) exclusion of females and c) male-only and SV analysis combined.

a.

b.

**Figure S1 a and b.** Dendrograms showing samples clustered based on Euclidean distance between gene expression profiles. A) labels are coloured by sex, b) labels coloured by group membership and twin pair. All individuals cluster as expected by sex, and all but one set of co-twins cluster together in pairs.

a. b.

c. d.

**Figure S2.** MDS plots showing similarity of samples based on BCV distance. Labels coloured by a. sex, b. group membership, c. case status, d. sequencing lane. Samples identified as outliers indicated with a cross. The variation projected on to the ﬁrst two dimensions appears to be largely accounted for by sex, group and case status.


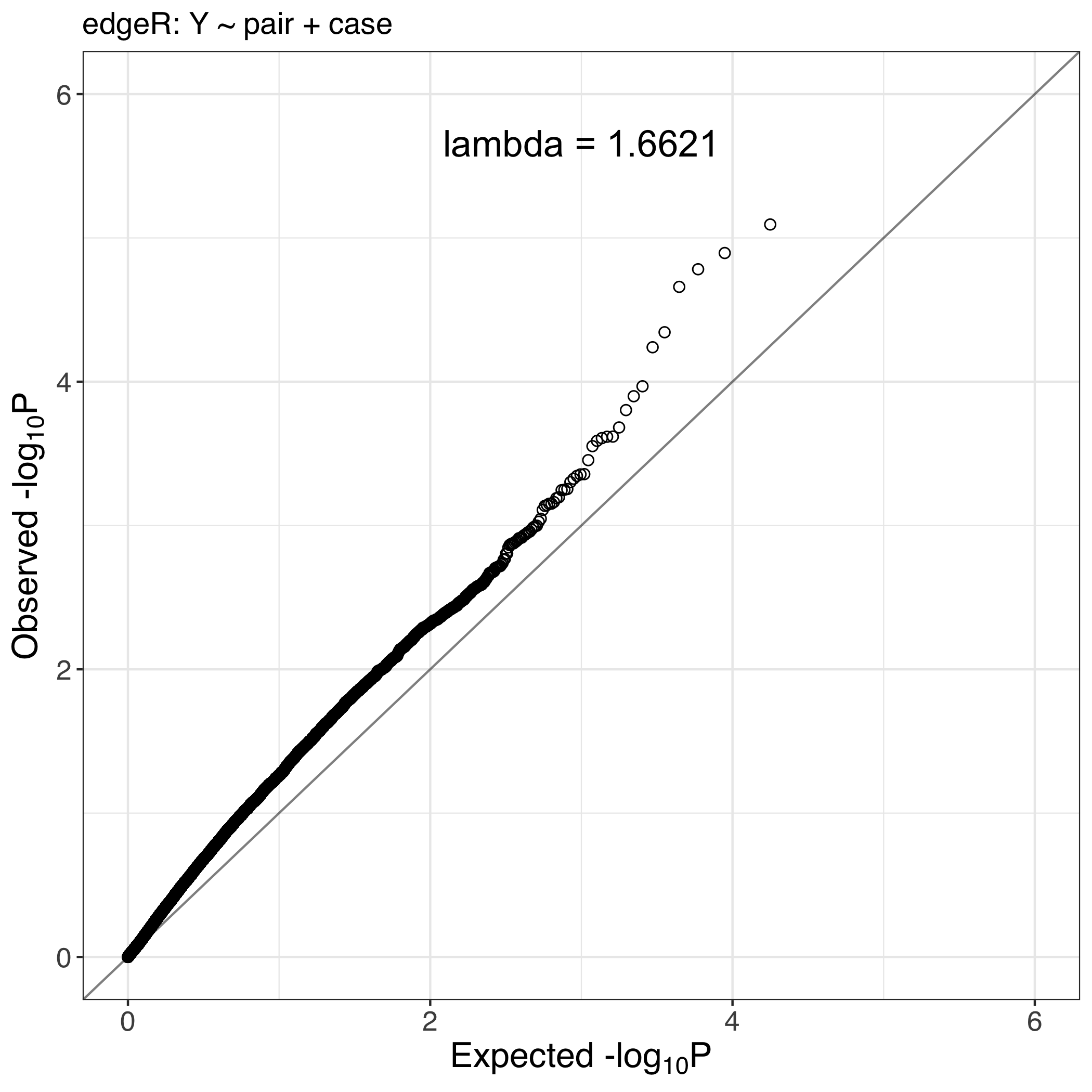

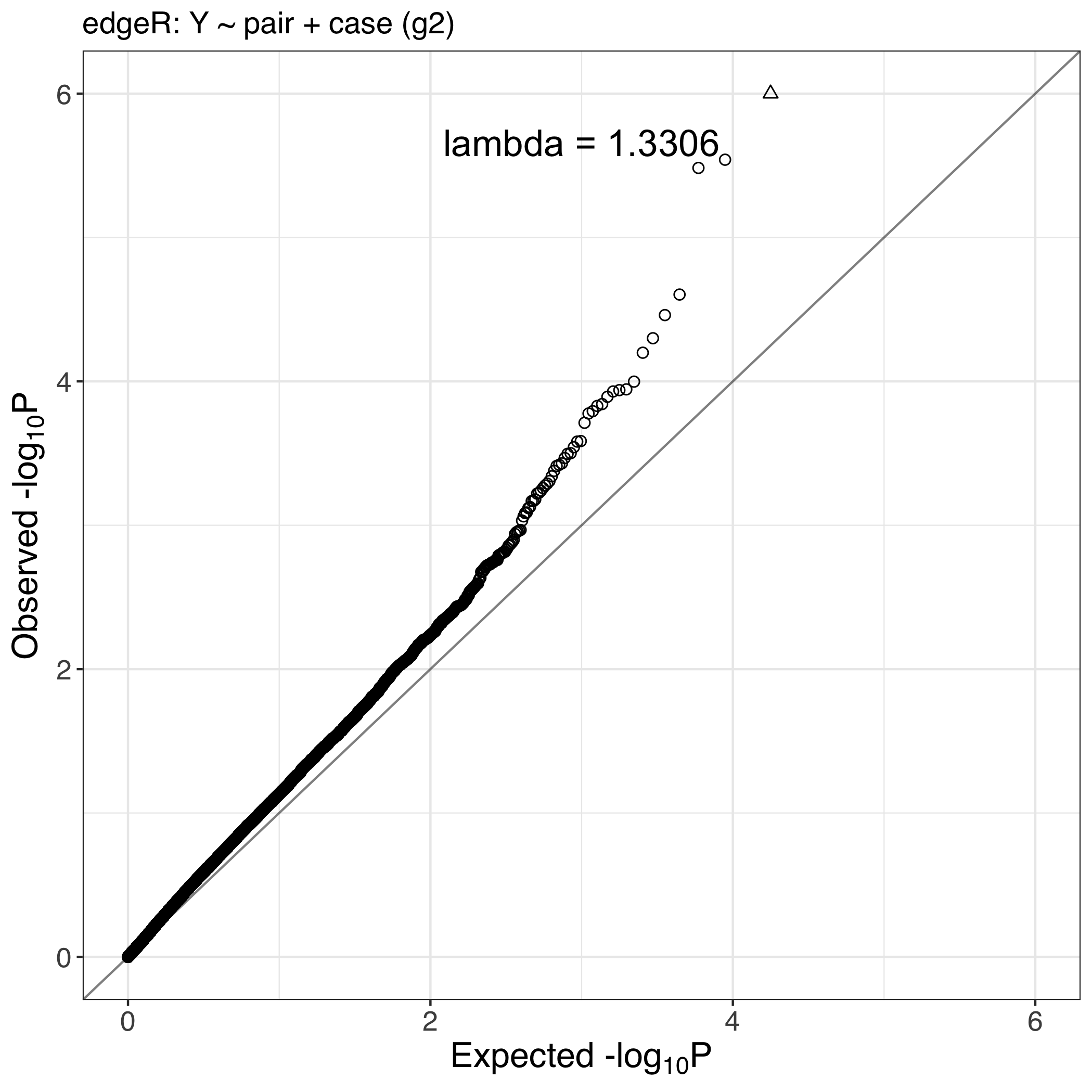


a. b.

**Figure S3 a, b**. QQ plots showing the observed vs theoretical distribution of p-values for a) discordant and b) case control analyses


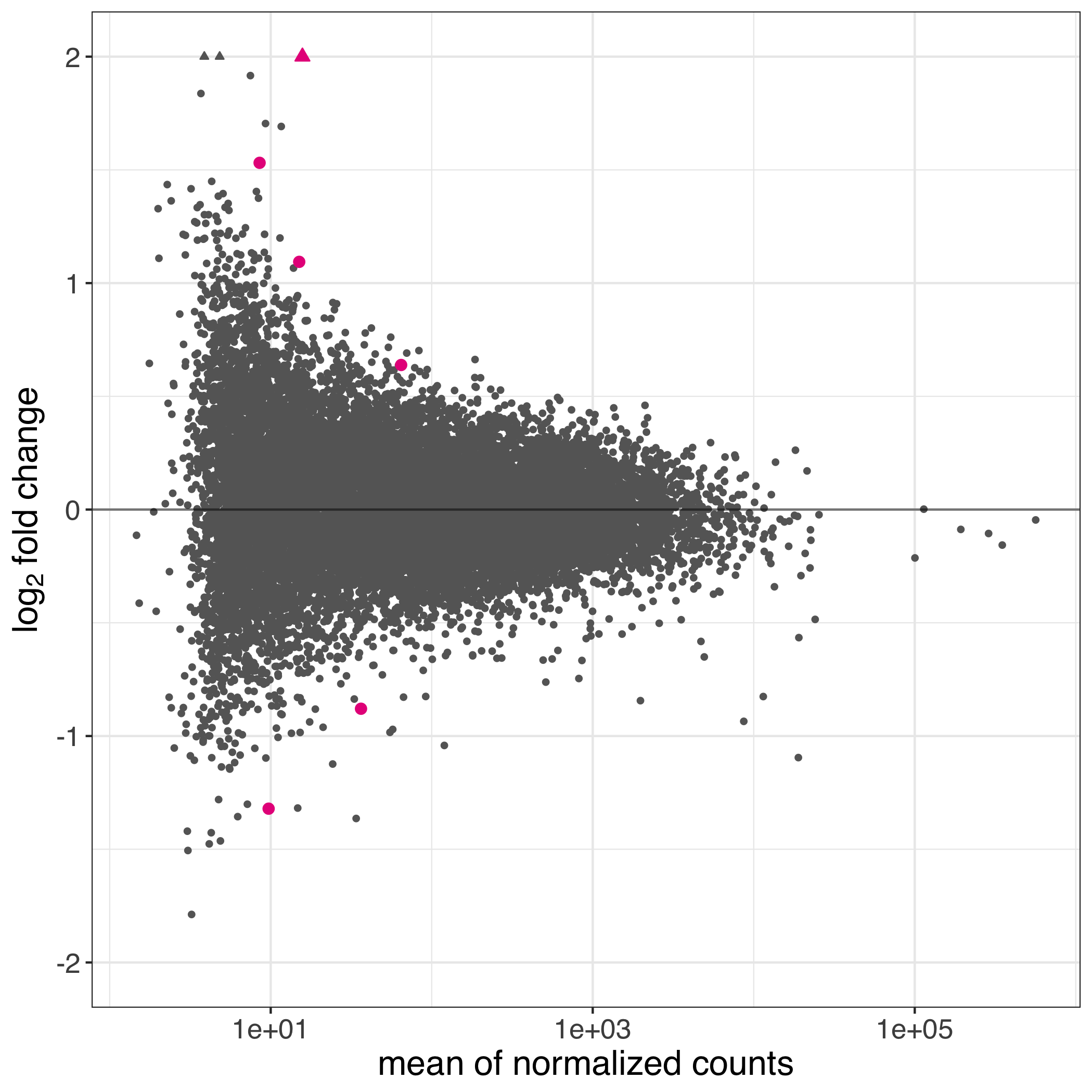

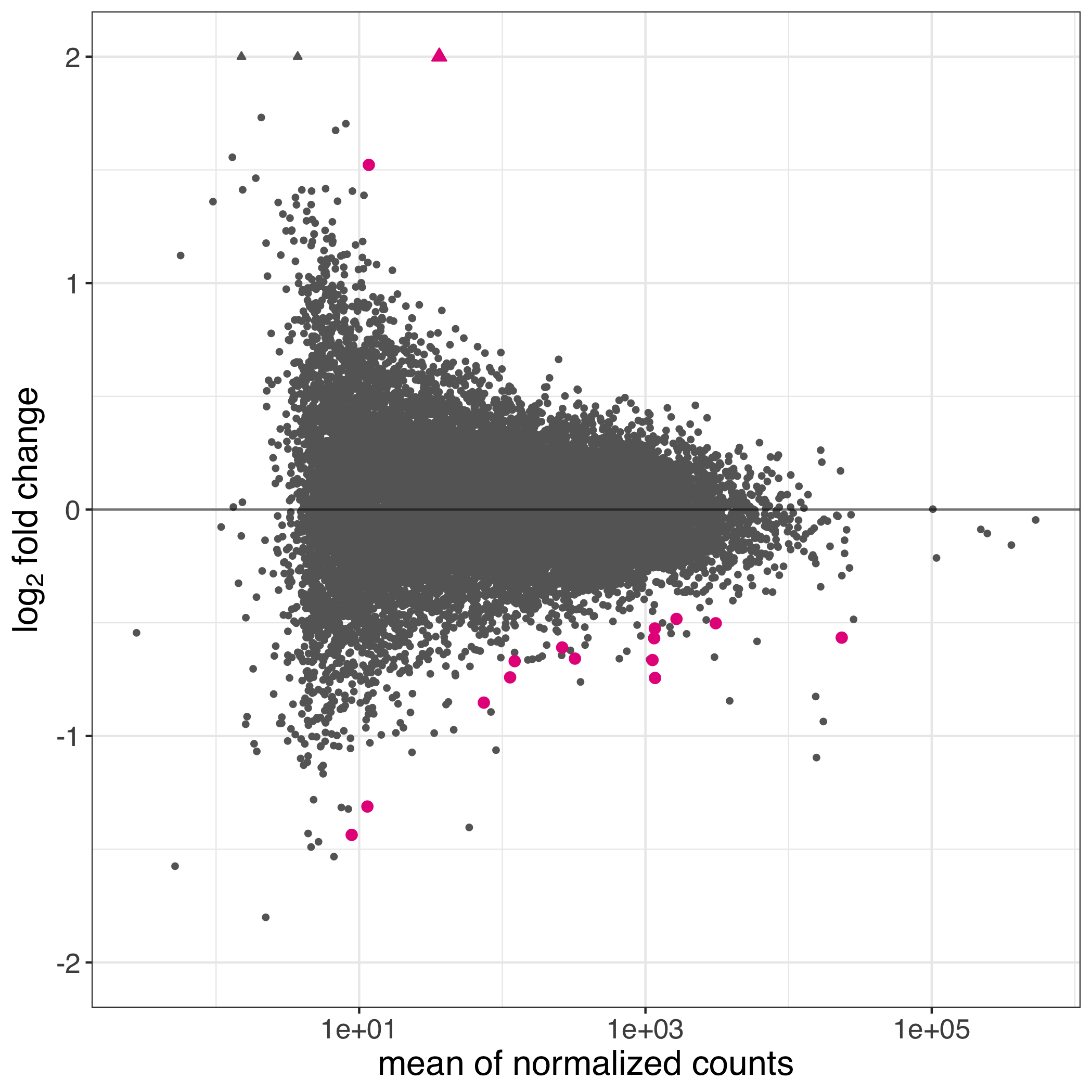


**Figure S4.** MA plots showing the relationship between logFC and mean count for a) discordant and b) case control analyses. Coloured points indicate genes passing FDR < 20%.

**
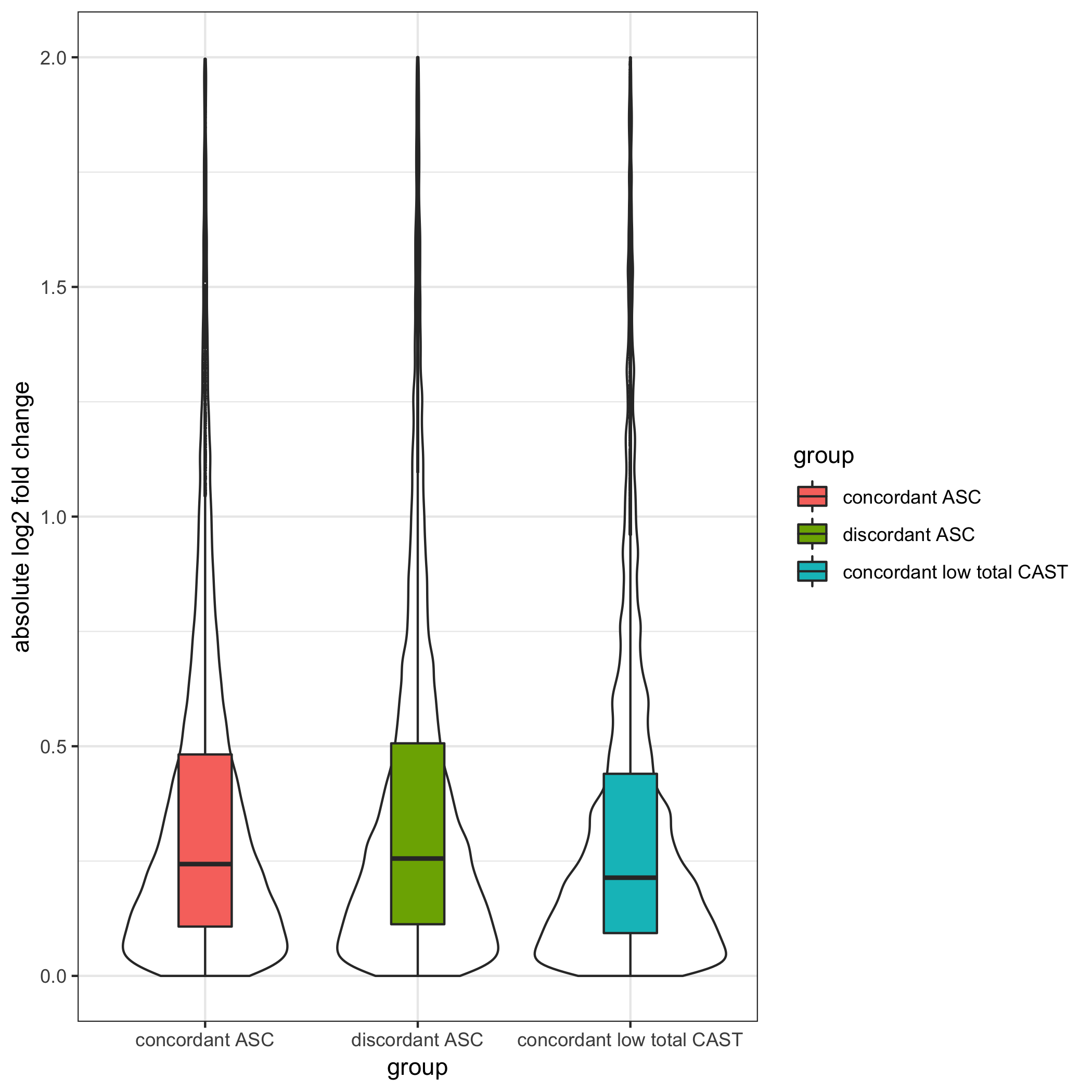
**

**Figure S5.** Distribution of within-pair log fold change for the different experimental groups. All three groups show different log FC distributions, with the concordant low ASC symptom twins showing the smallest mean expression differences.

**Figure S6.** Comparison of gene expression levels between ASC affected and unaffected cotwins for differentially expressed genes at FDR < 10% identified in the discordant pairs analysis: *IGHG4, EVI2A, SNORD15B*. CPM - counts per million.

**Figure S7.** Distributions of expression levels for differentially expressed genes at FDR < 10% identified in the case control analysis : *IGHG4, PRR13P5, DEPDC1B*, *ZNF50*1, stratified by study group and case status*.*  CPM - counts per million, cASC – concordant ASC, dASC - discordant ASC, cltC - concordant low total CAST.
